# From Robust Control to Cyber-Resilience: A Comprehensive Overview of the Polytopic Framework for Safety-Critical Systems

**DOI:** 10.3390/s26144647

**Published:** 2026-07-22

**Authors:** Souad Bezzaoucha Rebai

**Affiliations:** EIGSI-La Rochelle, MIA Laboratory, La Rochelle University, 26 rue de Vaux de Foletier, 17041 La Rochelle, France; souad.bezzaoucha@eigsi.org; Tel.: +33-(0)5-46-45-80-21

**Keywords:** sensors and actuators security, fault diagnosis, robust observers, multi-model systems, cyber-physical systems, anomaly detection, fault-tolerant control, event-triggered control

## Abstract

This overview paper highlights the idea that the polytopic approach is more than a modeling technique. It proposes a unified perspective in which polytopic representations constitute a conceptual bridge between complex system dynamics and convex analysis. From modeling to control, the polytope is not only limited to a geometric interpretation but is used as a methodological principle—an intelligent sensor system for structuring uncertainty, representing a nonlinear behaviors, and enabling decision-making, even for critical safety systems. Indeed, the polytopic approach should not be viewed merely as a convexification tool, but as a way of thinking about complexity, i.e., a structured methodology linking representation, uncertainty management, and control synthesis. For this purpose, this work focuses on safety-critical systems, i.e., cyber-physical systems operating under malicious cyber-attacks, where ensuring resilience has become a major challenge. The review highlights recent developments that extend classical polytopic approaches beyond robust control and toward cyber-resilient estimation and control, including false-data injection attack modeling, simultaneous state and attack estimation, resilient observer-based control, and event-triggered control under communication constraints. The paper also discusses how these developments position the polytopic framework with respect to recent advances in cyber-physical security and resilient control. Overall, the proposed perspective illustrates how polytopic representations have evolved into a unifying paradigm for addressing nonlinear dynamics, cyber-attacks, uncertainties, and network-induced constraints while enhancing the resilience, reliability, and operational safety of safety-critical cyber-physical systems.

## 1. Introduction

The increasing complexity of modern engineering systems has deeply transformed the way modeling, analysis, and control problems are formulated. Large-scale infrastructures, hydraulic networks, energy systems, transportation platforms, and cyber-physical architectures are characterized by nonlinear dynamics, parametric uncertainty, operational constraints, and evolving environments. Traditional linear control paradigms, although powerful and mathematically elegant, often struggle to capture the structural complexity inherent to such systems. At the same time, fully nonlinear approaches frequently lead to analytical complexity and computational burdens incompatible with real-time implementation. Among the available approaches, polytopic representations have progressively established themselves as a central paradigm, enabling the transformation of nonlinear and uncertain dynamics into convex formulations adapted to systematic analysis and control design.

Besides convex polytopic representations, recent research has investigated fully nonlinear control and estimation techniques for cyber-physical systems. Nonlinear Model Predictive Control (NMPC) [1,2], Markov decision-making processes [3], reinforcement learning [4], reduced order observers [5,6] and differential geometric approaches [7], for example, have demonstrated remarkable capabilities for handling severe nonlinearities and state/input constraints. These methods generally exploit the original nonlinear dynamics without approximation and may provide less conservative solutions than convex representations. However, they often require solving nonlinear optimization problems online, resulting in high computational complexity and limited scalability for large-scale or embedded safety-critical applications. Although these nonlinear methodologies have significantly advanced the control of cyber-physical systems, they generally require nonlinear optimization, intensive numerical computations, or large training datasets. In contrast, the polytopic framework provides an intermediate paradigm that transforms nonlinear dynamics into convex representations while preserving the main nonlinear characteristics of the original system. This enables the systematic synthesis of observers and controllers through convex LMI optimization, offering an attractive compromise between modeling fidelity, computational efficiency, and formal stability guarantees, particularly for safety-critical cyber-physical systems.

Indeed, the idea beyond polytopic thinking is that polytopes transform the nonlinearity into a convex structure. Nonlinearity is not eliminated; rather, it is reorganized into a geometry that is computationally exploitable. Indeed, polytopic representations have emerged as a powerful framework to bridge nonlinear modeling and convex control design. In these frameworks, the system dynamics are expressed as a convex combination of vertex subsystems, where the scheduling or interpolation parameters depend on measurable variables or bounded uncertainties. Such a formulation allows the use of convex analysis tools, particularly Linear Matrix Inequalities (LMIs), for stability and performance guarantees. However, the literature generally considers modeling, analysis, and control as distinct, though complementary, stages. The polytope appears either as a modeling toll or as a convex uncertainty set used during synthesis. By contrast, the idea of adopting a unified perspective, where representation and control are co-designed within a consistent polytopic framework, seems very appealing. By integrating recent advances in modeling, control, state estimation, and safety-oriented design, this paper seeks to provide a comprehensive and coherent overview of methodologies, applications and perspectives of polytopic and LMI-based techniques, from representation to control. These theoretical developments have been validated on critical application domains where safety, reliability, and performance are essential. The results illustrate how polytopic approaches can bridge advanced mathematical tools and practical engineering constraints.

It is worth emphasizing that the proposed polytopic framework relies on the Sector Nonlinearity (SNL) transformation, which yields an exact convex rewriting of the original nonlinear model rather than an approximation. As a result, the transformation introduces neither approximation nor estimation errors, and the resulting polytopic representation remains mathematically equivalent to the original nonlinear system over the prescribed operating domain. Consequently, the accuracy of the proposed framework depends exclusively on the fidelity of the original nonlinear model used to describe the physical process. This property constitutes one of the principal advantages of the SNL-based approach, as it preserves the original system dynamics while enabling the application of convex analysis and synthesis tools for observer and controller design.

Cyber-physical systems (CPSs) have become a major research focus in safety-critical domains due to their extensive deployment in transportation, energy, industrial automation, water management, and smart infrastructure. Consequently, the resilience of CPSs against cyber threats has emerged as a fundamental requirement for guaranteeing safe and reliable operation. Over the last decade, numerous studies have investigated control strategies capable of tackling various kinds of attacks, including false-data injection, denial-of-service (DoS), replay attacks, and communication-delay manipulation. Existing approaches for securing cyber-physical systems can generally be classified into passive and active defense mechanisms. Passive defenses aim at detecting, isolating, estimating, and mitigating attacks after they have occurred. Typical examples include observer-based residual generation, unknown-input observers, robust filtering, and resilient control. By contrast, active defenses proactively modify the system behavior to invalidate the attacker’s knowledge or increase attack detectability. In particular, Xu [8] proposed a switching-based MTD framework in which multiple controllers are switched according to a stochastic policy, preventing attackers from accurately identifying the system dynamics while maintaining closed-loop stability. Although the terms robustness, resilience, safety, and security are sometimes used interchangeably in the literature, they describe complementary properties of cyber-physical systems. Robustness refers to the capability of maintaining prescribed performance despite modeling uncertainties, parameter variations, and external disturbances. Resilience extends this concept by enabling the system to anticipate, detect, withstand, respond to, and recover from adverse events, including malicious cyber-attacks. Likewise, safety concerns the prevention of unacceptable physical harm, whereas security addresses protection against intentional threats targeting system integrity, availability, and confidentiality. In cyber-physical systems, these concepts are inherently interconnected: robust control contributes to safe operation under uncertainties, while resilient control combines robustness with monitoring, estimation, and recovery mechanisms to preserve safety despite security breaches. This work illustrates the increasing interest in combining active defense with resilient control to enhance cyber-security against stealthy false-data injection attacks. Although active defense significantly enhances cyber-resilience, it is generally complementary rather than alternative to model-based estimation. The proposed approach instead focuses on exploiting the polytopic representation to simultaneously estimate the system states and the attack signals. Consequently, the proposed observer-based methodology is complementary to active defense mechanisms and could naturally be integrated within a layered cyber-resilience architecture combining proactive protection with robust state and attack estimation.

Unlike conventional control systems, modern Cyber-Physical Systems (CPSs) extend conventional control systems by integrating sensing, computation, communication, and control over interconnected cyber infrastructures, thereby introducing cyber-security challenges that are generally absent or significantly less pronounced in traditional, non-networked control architectures. The large number of distributed sensors, actuators, communication channels, and interacting subsystems further increases the complexity of ensuring secure operation. Maintaining performance under adversarial conditions therefore requires the development of advanced monitoring, estimation, and feedback-control strategies capable of detecting abnormal-non nominal behaviors while preserving operational continuity and equipment safety. Indeed, recent research has increasingly explored the integration of resilient control with learning-based estimation, distributed architectures, and data-driven anomaly detection; see [9,10,11,12,13,14,15] for more details. While these approaches significantly improve adaptability, the need for mathematically rigorous frameworks providing formal stability and performance guarantees remains an active research challenge. In this context, the polytopic paradigm offers a complementary model-based foundation that can naturally accommodate these emerging developments.

Within this context, this work focuses on the analysis of stability, state estimation, and control problems for CPSs operating under malicious data-manipulation attacks, i.e., data deception attacks. Particular attention is devoted to model-based attack detection, identification, and isolation techniques, which exploit mathematical descriptions of system dynamics to generate diagnostic information and detect the presence of abnormal or adversarial activities. Based on the process models, these approaches provide a systematic framework for detecting and estimating system behaviors. Indeed, from a methodological perspective, the proposed framework differs from conventional resilient observer paradigms such as Unknown Input Observers (UIOs) and interval observers. UIO-based techniques typically rely on decoupling conditions that may restrict their applicability to specific system structures, whereas interval observers focus on computing guaranteed upper and lower bounds of the system states under bounded uncertainties. In contrast, the proposed polytopic framework provides a unified representation in which nonlinearities, parameter variations, uncertainties, and multiplicative cyber-attacks are embedded within a common convex formulation, enabling the simultaneous synthesis of resilient observers and controllers through a single design methodology. The emphasis of the present work is therefore on the unifying capability of the polytopic framework rather than on benchmarking individual resilient estimation paradigms.

The proposed design of resilient estimation and control schemes is based on the well-established approach of fault diagnosis. Classical fault-detection and isolation (FDI) methods rely on comparing measured outputs with model-based predictions and generating residual signals that reflect deviations from nominal behavior. These residuals are subsequently evaluated to determine whether a fault or anomaly has occurred. However, when a large number of potential failure scenarios must be considered, the construction and analysis of dedicated residuals for every fault mode become computationally demanding and often impractical. To address these limitations, recent research has been successfully applied to intelligent transportation systems, where adaptive thresholding techniques have been introduced to improve sensitivity and reduce detection delays.

It is important to highlight the difference between cyber-attacks and physical attacks. Indeed, the vulnerability of CPSs is due to both. Cyber-attacks primarily target information-processing and communication infrastructures by compromising data integrity, confidentiality, or availability. Physical attacks, often referred to as kinetic attacks, directly affect hardware components, sensors, actuators, or critical infrastructures through intentional manipulation or damage. Sensors are particularly exposed because they constitute the primary interface between the physical environment and the cyber layer. As a result, corrupted measurements can significantly degrade the performance of estimation, monitoring, and control algorithms, especially when adversaries exploit both cyber and physical vulnerabilities simultaneously.

Although closely related, cyber-security and cyber-physical security address different objectives. Traditional cyber-security focuses mainly on protecting digital assets and communication networks by ensuring data confidentiality, integrity, and authenticity. Cyber-physical security extends these objectives by incorporating the physical dynamics and operational constraints of the controlled process. Its primary goal is to preserve system functionality, safety, and availability even when security mechanisms at higher information-technology layers have been compromised. Consequently, cyber-physical security does not merely attempt to prevent attacks but also seeks to identify their effects through the observation of abnormal physical behaviors and dynamic inconsistencies.

Another important distinction lies in the underlying detection mechanisms. Conventional cyber-security solutions typically monitor network traffic, communication protocols, and information exchanges to identify suspicious activities. In contrast, cyber-physical security exploits process knowledge and system dynamics to detect deviations in physical behavior that may indicate either cyber or physical attacks. This specificity enables the detection of a broader range of threats, including those that remain invisible to network-based monitoring tools. Therefore, cyber-physical security can be regarded as an additional and complementary defense layer that enhances overall system resilience.

The growing sophistication of adversarial threats highlights the need for integrated approaches combining attack detection, resilient estimation, and secure control design. Such methodologies are essential for ensuring continuous operation, maintaining system integrity, and guaranteeing safe performance in the presence of both accidental faults and intentional attacks. As CPSs continue to play a critical role in modern infrastructures, the development of robust and resilient control architectures remains a key research challenge.

To address this challenge, one effective approach is the Model-Based Attack Detection, Identification, and Isolation framework, which relies on a mathematical model of the system to facilitate attack detection, isolation, and identification. The required model is generally obtained either from first-principles modeling or from system identification based on operational data. Once an accurate dynamic model of the process has been established, observer-based monitoring schemes constitute one of the most widely adopted tools for attack detection and localization. In the literature, attack isolation is generally achieved through two principal paradigms. The first relies on multiple observers operating simultaneously, where each observer (e.g., Kalman filters, Luenberger observers) is specifically designed to react to a particular attack scenario or system component [16,17,18]. By analyzing the residual signals generated by the observer bank, the compromised element can be identified. The second paradigm follows a hierarchical monitoring architecture, in which detection is initially performed at the global system level and progressively refined toward subsystems, sensors, actuators, or communication channels through a top-down diagnostic process [19,20,21]. While both approaches have demonstrated their effectiveness in a variety of applications, hierarchical strategies may suffer from increased detection latency due to the sequential nature of the localization procedure.

From the perspective of system dynamics and control, malicious attacks can often be interpreted as unknown disturbances intentionally introduced into the process. However, the growing sophistication of cyber threats has revealed the limitations of conventional fault-detection frameworks. In many practical situations, attack signals enter the system through nonlinear mechanisms, exhibit time-varying characteristics, or are deliberately designed to remain undetected. Consequently, the analysis of cyber-physical attacks requires more advanced methodologies than those traditionally employed in fault diagnosis.

To address these challenges, increasing attention has been devoted to the integration of nonlinear estimation techniques, robust control theory, and data-driven methods. Such hybrid approaches seek to improve detection capabilities while reducing the conservatism often associated with classical model-based formulations. In particular, resilient state estimation and robust control design have emerged as promising directions for enhancing the security and reliability of cyber-physical systems operating in adversarial environments. Recent contributions include robust state-estimation techniques formulated in the $L_{2}$ framework for networked systems affected by deception attacks, event-triggered control architectures and resilient-control methodologies that jointly address performance, robustness, and security requirements.

Beyond attack detection, recent research has increasingly focused on the design of resilient control architectures capable of maintaining acceptable performance despite ongoing attacks. In this context, robust control techniques provide a natural framework for handling uncertainties and adversarial perturbations simultaneously. Particular attention has been devoted to nonlinear systems, where fuzzy-model-based formulations and attack-resilient estimation strategies have been employed to achieve stabilization and performance guarantees under malicious conditions. The fundamental objective of resilient estimation and control is not merely to detect attacks but also to mitigate their consequences, enabling the system to continue operating safely even when measurements, communication channels, or control inputs have been compromised.

More generally, cyber-security and cyber-resilience can be viewed as a multi-stage process encompassing several complementary functions. Detection aims at determining whether an attack has occurred; isolation seeks to identify the affected components, such as sensors, actuators, or communication nodes; identification and estimation evaluate the characteristics of the attack, including its location, magnitude, and impact on system behavior; and finally, resilience mechanisms strive to preserve system operability, safety, and availability through attack accommodation and reconfiguration strategies. Depending on the severity of the incident, these mechanisms may maintain nominal performance or ensure continued operation under degraded conditions.

While robust control strategies provide satisfactory performance and stability in the presence of uncertainties and disturbances, their implementation often relies on periodic sensing and actuation. To reduce communication and computational burdens, event-based control has emerged as an attractive alternative. In our own previous papers [22,23,24,25], the polytopic Takagi–Sugeno approach was already applied for state and stealthy attack estimation where an event-based approach was considered. The proposed event-triggered control strategy, based on the same idea applied to the actuator saturation problem (usually treated as external perturbations or managed through ad hoc compensation strategies), is a decomposition-based modeling approach allowing such structured nonlinearities to be incorporated directly into the convex polytopic framework. Beyond classical control applications, the approach was extended to emerging domains, i.e., cyber-security and cyber-physical systems, where malicious attacks and data integrity issues introduce new forms of disturbances. By reformulating attack and intrusion models within a polytopic or multiple-model setting, the proposed work provided one of the earliest bridges between robust control theory and security-aware system design.

This work investigates the control of cyber-physical systems operating in safety-critical environments by reviewing two complementary strategies for enhancing resilience against malicious cyber-attacks and communication constraints. Rather than contrasting robustness with resilience, the title From Robust Control to Cyber-Resilience reflects the natural evolution of the proposed polytopic framework. Robust control constitutes the theoretical cornerstone for handling uncertainties and disturbances, whereas cyber-resilience extends this foundation by incorporating attack estimation, resilient control, and communication-aware mechanisms capable of maintaining safe system operation under adversarial conditions. Within this perspective, the present review illustrates how these concepts complement one another and have progressively evolved into a unified framework for resilient cyber-physical systems. The first strategy focuses on the development of attack-resilient control frameworks capable of preserving stability and performance despite the presence of adversarial disturbances. The objective is twofold: to attenuate the impact of attacks on the system dynamics through robust control design and to provide reliable state and attack estimation mechanisms that facilitate detection, isolation, and diagnosis procedures. By jointly addressing control and monitoring objectives, the proposed framework aims to guarantee secure control even under hostile conditions.

The second strategy explores the use of event-triggered control and observation schemes for nonlinear cyber-physical systems. Unlike conventional periodic implementations, event-based approaches are well-adapted for network-induced limitations such as bandwidth restrictions, communication delays, and limited computational resources. These considerations are particularly relevant in large-scale networked systems, where continuous information exchange may become impractical or impact the computational burden. Achieving a realistic representation of safety-critical systems requires the incorporation of nonlinear dynamics throughout the entire design process, including modeling, estimation, diagnosis, and control synthesis. However, the resulting nonlinear models often lead to high computational complexity and increased communication requirements. To lighten these limitations, an event-triggering mechanism is integrated with the robust control architecture, allowing control updates to be transmitted only when predefined conditions are satisfied. Such a strategy significantly reduces communication and computation loads while maintaining the desired control performance.

Based on this principle, a co-design framework is proposed in which observer synthesis and event-triggered control design are addressed simultaneously. The resulting architecture combines a resilient state-and-attack observer with an event-based controller, leading to a unified monitoring and control structure. By considering the closed-loop augmented dynamics, sufficient conditions are established to ensure stability and robustness in the presence of attacks and network constraints. The proposed synthesis methodology relies on Lyapunov-based stability analysis and convex optimization techniques formulated in terms of Linear Matrix Inequalities (LMIs), from which the observer and controller gains are systematically derived.

Unlike a conventional research article introducing a single methodological contribution, this paper aims to provide a unified retrospective of a coherent body of research developed over the past several years. Its objective is to identify the common theoretical foundations, methodological evolution, and conceptual links between previously published contributions, thereby offering a comprehensive perspective on the development of the polytopic framework and its applications to increasingly complex control problems.

The remainder of this work is organized as follows. First, the polytopic framework is introduced as a unified modeling and analysis tool for a broad class of nonlinear, uncertain, parameter-varying, and cyber-physical systems. Based on this framework, a robust output-feedback $H_{\infty }$ observer-based Takagi–Sugeno (T-S) control strategy is developed to ensure stability and disturbance attenuation in the presence of system uncertainties. Then, an event-triggered control scheme is investigated as an alternative solution aimed at reducing communication and computational demands while preserving the desired control performance. Both control strategies are then extended to cyber-physical systems operating under data-deception attacks, where malicious alterations of transmitted information may compromise system integrity and performance. An illustrative example, validated by rigorous stability and performance analyses, is presented for the proposed approaches in Section 6. Finally, the conclusion and perspectives is detailed in the final section.

## 2. From Robustness to Cyber-Resilience: Evolution of Control Strategies for Cyber-Physical Systems

The increasing interconnection between physical processes, communication networks, and embedded computing has profoundly transformed modern control systems into cyber-physical systems (CPSs). While this integration has enabled higher levels of autonomy, intelligence, and connectivity, it has also introduced new vulnerabilities arising from cyber-attacks, communication failures, and coordinated malicious actions. Consequently, the objectives of control engineering have progressively evolved from ensuring robustness against uncertainties toward guaranteeing resilient operation under intentional adversarial conditions.

Classical robust control theory was originally developed to guarantee closed-loop stability and prescribed performance despite modeling errors, parameter uncertainties, and external disturbances. Techniques such as $H_{\infty }$ control, sliding-mode control, adaptive control, model predictive control, and LPV/Takagi–Sugeno polytopic approaches have provided mathematically rigorous tools for designing controllers capable of maintaining acceptable performance under bounded uncertainties. These methods have become fundamental in many safety-critical applications, including aerospace systems, autonomous vehicles, industrial automation, and energy systems, where reliability and stability are essential requirements.

However, the emergence of cyber-physical systems has revealed that classical robustness alone is no longer sufficient. Unlike conventional disturbances, malicious cyber-attacks are intentional, adaptive, and specifically designed to exploit system vulnerabilities while remaining difficult to detect. False-data injection attacks, replay attacks, denial-of-service attacks, actuator and sensor manipulations, and stealthy attacks may simultaneously affect both the physical dynamics and the communication infrastructure. These threats can significantly degrade estimation accuracy and control performance while potentially remaining invisible to controllers designed for uncertainty rejection.

These challenges have motivated the emergence of cyber-resilient control, whose objective extends beyond maintaining robustness to disturbances. A resilient control architecture aims to preserve acceptable system performance before, during, and after an attack by integrating complementary functionalities such as attack detection, fault isolation, secure state estimation, resilient control reconfiguration, and recovery mechanisms. Rather than considering robustness and resilience as competing concepts, resilience should be viewed as a natural extension of robust control. Robustness provides the mathematical foundation required to guarantee stability under uncertainties, whereas resilience enriches this foundation with decision-making, monitoring, and recovery capabilities specifically designed to address intelligent adversaries.

Recent research has therefore increasingly focused on integrating resilient control with complementary paradigms. Distributed and decentralized control architectures improve scalability and fault tolerance for large-scale networked CPSs and multi-agent systems. Learning-assisted estimation and adaptive control techniques provide the ability to cope with evolving operating conditions and previously unseen attack strategies by exploiting online data. Similarly, data-driven anomaly detection and machine learning algorithms have demonstrated promising capabilities for identifying complex or stealthy attacks that may be difficult to characterize using purely analytical models; see Refs. [9,10,11,12,13,14,15] for more details. More recently, physics-informed machine learning has emerged as a particularly attractive direction, combining the interpretability and formal guarantees of model-based approaches with the adaptability of data-driven methods. Rather than replacing classical control theory, these emerging techniques complement rigorous observer- and model-based frameworks by enhancing adaptability while preserving stability and robustness guarantees.

Within this context, the polytopic framework reviewed in this article follows this methodological evolution. Initially developed to address nonlinear systems, parameter uncertainties, and robust observer-based control, it has progressively evolved toward the joint estimation of system states and cyber-attacks, resilient observer–controller co-design, and communication-aware event-triggered strategies. Consequently, the transition from robust control to cyber-resilience should not be interpreted as a conceptual shift away from robustness, but rather as the progressive enrichment of robust control methodologies to meet the challenges posed by modern cyber-physical systems. This evolution naturally positions the proposed framework as a bridge between established model-based control theory and emerging intelligent resilient architectures, providing a rigorous foundation upon which future distributed, adaptive, and learning-enhanced resilient control strategies can be developed.

## 3. The Polytopic Modeling of CPS

This section presents how the polytopic frameworks are used as a unifying approach for addressing nonlinear, uncertain, parameter-varying systems.

As already stated, the main idea of the polytopic approach is to approximate or transform nonlinear system dynamics into a convex combination of linear submodels. This representation allows one to use powerful tools developed for linear systems while capturing essential nonlinear behaviors. Such formulations are particularly attractive for control synthesis, state estimation, and stability analysis, especially when dealing with systems exhibiting strong nonlinearities, parameter variations, or multi-operating regimes.

While the literature has extensively addressed stability analysis, control design, and state estimation within the Takagi–Sugeno, multiple models, and polytopic representations, these contributions largely focused on systems with either constant or slowly varying parameters. In this context, one of the first structured methodologies providing a framework to explicitly handle time-varying parameters systems within a rigorous convex framework was introduced in our work in [25]. This approach provided a systematic foundation for extending convex control and estimation techniques to a broader class of nonlinear and parameter-varying systems.

In this section, the proposed framework is used to model Cyber-Physical Systems (CPSs). Different from traditional control systems, the tight integration of physical and cyber-components, and the occurrence of various malicious attacks, pose nontrivial challenges to the performance analysis and the design of state estimators or filters. These systems typically exhibit hybrid dynamics, uncertainties, and nonlinear behaviors, making them particularly suitable for polytopic and multiple-model representations. An exact reformulation is proposed thanks to the sector nonlinear transformation approach (SNT). In the following, the modeling of Cyber-Physical Systems using the polytopic framework is presented.

### 3.1. Polytopic Modeling of Attacked Systems

This section examines cyber-physical systems subjected to false-data injection attacks and investigates their analysis, monitoring, and control within the polytopic framework. Among the various model-based methodologies proposed in the literature, the polytopic approach has emerged as a particularly attractive paradigm due to its ability to unify the treatment of nonlinearities, uncertainties, parameter variations, and cyber-security challenges within a common convex optimization framework. Such an advantage is especially valuable in cyber-physical systems, where the strong interaction between physical processes, computational units, communication networks, and control mechanisms gives rise to highly complex and heterogeneous dynamics.

The analysis and control of CPSs are further complicated by nonlinear behaviors, uncertain parameters, and the presence of malicious disturbances. To address these challenges, the polytopic framework represents the system dynamics as a convex combination of multiple local linear models, each associated with a particular operating regime. The interpolation between these local descriptions is governed by scheduling functions linked to varying parameters, enabling a smooth transition across operating conditions while preserving the dominant nonlinear characteristics of the original process.

One of the major advantages of this representation lies in its ability to bridge nonlinear system modeling and linear control theory. By transforming a complex nonlinear system into a convex set of linear submodels, the polytopic formulation enables the use of powerful and well-established analysis and synthesis tools developed for linear systems. In particular, it provides a convenient framework for the design of state observers, fault-diagnosis schemes, and resilient controllers while maintaining a faithful representation of the system dynamics.

Moreover, the convex structure inherent to polytopic models naturally leads to optimization-based formulations, allowing stability, robustness, and performance requirements to be expressed as Linear Matrix Inequality (LMI) conditions. This feature has contributed significantly to the adoption of polytopic techniques in critical control applications, where rigorous guarantees on system behavior are required. Consequently, the polytopic framework offers a rigorous formulation for the analysis, estimation, monitoring, and secure control of cyber-physical systems operating under adverse and potentially hostile conditions.

#### 3.1.1. Polytopic Modeling of CPS Under Data Deception Attacks

To represent the impact of cyber-attacks on both actuators and sensors, a unified polytopic representation is considered. The proposed formulation embeds actuator and sensor attacks directly into the system dynamics through a set of time-varying parameters, allowing the attack effects to be analyzed within the same modeling framework as the nominal system dynamics. Such a representation is particularly attractive for cyber-physical systems, as it enables the simultaneous treatment of nonlinearities, parameter variations, uncertainties, and malicious disturbances using convex optimization tools.

Let us consider the nonlinear cyber-physical system described by (2). The vector of time-varying parameters $\theta \left(t\right)\in R^{n}$ is introduced to characterize the attack signals affecting the system and is partitioned as:

(1) $$\theta \left(t\right)=\left[\begin{array}{c} \theta^{u}\left(t\right)\;\theta^{y}\left(t\right) \end{array}\right],$$

where $\theta^{u}\left(t\right)\in R^{n_{\theta_{u}}}$ and $\theta^{y}\left(t\right)\in R^{n_{\theta_{y}}}$ denote the actuator- and sensor-related attack parameters, respectively, with $n=n_{\theta_{u}}+n_{\theta_{y}}$. The vectors $x\left(t\right)\in R^{n_{x}}$, $u\left(t\right)\in R^{n_{u}}$, and $y\left(t\right)\in R^{m}$ represent the state, control input, and measured output respectively.

The nonlinear dynamics are expressed through a polytopic decomposition involving *r* local linear models. Such a representation can be systematically obtained using the Sector Nonlinearity Transformation (SNT), which converts nonlinear dynamics into a convex combination of linear submodels. Detailed works of SNT-based polytopic models can be found in [23,25,26].

The resulting system is described by:

(2) $$\begin{array}{ccc} \dot{x}\left(t\right) & =\sum_{i=1}^{r}\mu_{i}\left(x\left(t\right)\right)\left(A_{i}x\left(t\right)+B_{i}\left(t\right)u\left(t\right)\right),\;y\left(t\right) & =C(t)x(t), \end{array}$$

where $\mu_{i}\left(x\left(t\right)\right)$ are the weighting functions satisfying the convex-sum property and ensuring smooth interpolation between the local models.

To explicitly model the effects of cyber-attacks acting on the actuation and sensing layers, the input and output matrices are parameterized as follows:

(3) $$\begin{array}{ccc} B_{i}\left(t\right) & =B_{i}+\sum_{j=1}^{n_{\theta_{u}}}\theta_{j}^{u}\left(t\right),{\bar{B}}_{ij},\;C\left(t\right) & =\left(I_{m}+F\left(t\right)\right)C, \end{array}$$

where $B_{i}$ and ${\bar{B}}_{ij}$ are known constant matrices of compatible dimensions. The coefficients $\theta_{j}^{u}\left(t\right)$ are unknown time-varying parameters representing multiplicative actuator attacks.

Similarly, the sensor attack mechanism is captured through the matrix:

(4) $$F\left(t\right)=\mathrm{diag}\left(\theta^{y}\left(t\right)\right),$$

where each diagonal entry corresponds to the attack level affecting a particular sensor channel. F(t) can be rewritten as:

(5) $$F\left(t\right)=\sum_{j=1}^{n_{\theta_{y}}}\theta_{j}^{y}\left(t\right)F_{j},$$

where $n_{\theta_{y}}=m$ and $F_{j}\in R^{m\times m}$ denotes the canonical diagonal matrix whose only nonzero element is located at position (j,j). The coefficients $\theta_{j}^{y}\left(t\right)$ therefore quantify the intensity of the multiplicative attacks acting on the corresponding sensors.

This formulation provides a unified description of actuator and sensor attacks within a polytopic framework and establishes a suitable basis for the following development of attack estimation and resilient control strategies. By embedding malicious perturbations directly into the model structure, the proposed representation enables the synthesis of observers and controllers capable of explicitly accounting for the presence of cyber-attacks while preserving stability and performance guarantees.

**Remark** **1.** *It is important to highlight that false-data injection attacks are commonly modeled in the literature as additive perturbations affecting transmitted sensor measurements or actuator commands. Such formulations are particularly suitable for representing injected biases, offsets, replayed signals, or corrupted communication packets.*

*In the present work, a multiplicative representation is deliberately adopted in order to describe cyber-attacks that modify the effectiveness or scaling of the sensing and actuation channels. From a physical viewpoint, this formulation models malicious alterations such as compromised sensor calibration factors, gain manipulation, partial loss or amplification of actuator authority, or coordinated scaling attacks affecting the input–output behavior of the system. Consequently, the attack is represented as a time-varying modification of the system matrices rather than as an external additive disturbance.*

*Although this mathematical formulation resembles multiplicative fault models commonly used in classical fault diagnosis, the underlying physical interpretation is fundamentally different. In fault diagnosis, multiplicative parameters generally represent accidental component degradations, efficiency losses, aging effects, or calibration drifts. Here, the parameters $\theta_{j}^{u}\left(t\right)$ and $\theta_{j}^{y}\left(t\right)$ introduced in this work represent intentional and adversarial cyber manipulations targeting the sensing and actuation channels.*

*This representation is particularly attractive within the proposed polytopic framework, since bounded multiplicative attack parameters can naturally be embedded as scheduling variables through the Sector Nonlinearity Transformation (SNT). Consequently, the attacked nonlinear system can be reformulated as an exact convex polytopic model, allowing the simultaneous design of resilient observers and controllers using LMI-based optimization techniques while explicitly accounting for cyber-attacks.*

#### 3.1.2. Modeling of Adversarial Perturbations Within the Polytopic Framework

The multiplicative false-data injection attacks affecting the actuators are characterized by the scheduling parameters $\theta_{j}^{u}\left(t\right)$. Although these attack signals are unknown, they are assumed to remain bounded within known intervals, i.e.,

$$\theta_{j}^{u}\left(t\right)\in \left[{\theta_{j}^{u}}^{\;2},{\theta_{j}^{u}}^{\;1}\right].$$

This assumption is commonly adopted in robust estimation and control frameworks, as it allows the attack effects to be represented as admissible parameter variations. By exploiting the Sector Nonlinearity Transformation (SNT), each parameter $\theta_{j}^{u}\left(t\right)$ can be expressed as a convex combination of its extreme values, thereby enabling its incorporation into the polytopic model representation. More precisely, the following decomposition holds:

(6) $$\theta_{j}^{u}\left(t\right)={\tilde{\mu }}_{j}^{1}\left(\theta_{j}^{u}\left(t\right)\right){\theta_{j}^{1}}^{u}+{\tilde{\mu }}_{j}^{2}\left(\theta_{j}^{u}\left(t\right)\right){\theta_{j}^{2}}^{u}$$

with

(7) $$\left\{\begin{array}{ccc} {\tilde{\mu }}_{j}^{1}\left(\theta_{j}^{u}\left(t\right)\right) & = & \frac{\theta_{j}^{u}\left(t\right)-{\theta_{j}^{2}}^{u}}{{\theta_{j}^{1}}^{u}-{\theta_{j}^{2}}^{u}}\\ {\tilde{\mu }}_{j}^{2}\left(\theta_{j}^{u}\left(t\right)\right) & = & \frac{{\theta_{j}^{1}}^{u}-\theta_{j}\left(t\right)}{{\theta_{j}^{1}}^{u}-{\theta_{j}^{2}}^{u}} \end{array}\right.$$

$${\tilde{\mu }}_{j}^{1}\left(\theta_{j}^{u}\left(t\right)\right)+{\tilde{\mu }}_{j}^{2}\left(\theta_{j}^{u}\left(t\right)\right)=1,\;\forall t$$

This decomposition transforms the unknown attack parameters into a convex representation that can be naturally integrated into the overall polytopic model. As a result, attack estimation, fault diagnosis, and resilient controller synthesis can be formulated within a common Linear Matrix Inequality (LMI)-based framework while preserving the original nonlinear dependence on the attack signals.

In a similar manner, the sensor data deception, or false data injection are modeled thanks to the time-varying parameters $\theta_{j}^{y}\left(t\right)$, such that:

(8) $$\theta_{j}^{y}\left(t\right)={\bar{\mu }}_{j}^{1}\left(\theta_{j}^{y}\left(t\right)\right){\theta_{j}^{1}}^{y}+{\bar{\mu }}_{j}^{2}\left(\theta_{j}^{y}\left(t\right)\right){\theta_{j}^{2}}^{y}$$

with

(9) $$\left\{\begin{array}{ccc} {\bar{\mu }}_{j}^{1}\left(\theta_{j}^{y}\left(t\right)\right) & = & \frac{\theta_{j}^{y}\left(t\right)-{\theta_{j}^{2}}^{y}}{{\theta_{j}^{1}}^{y}-{\theta_{j}^{2}}^{y}}\\ {\bar{\mu }}_{j}^{2}\left(\theta_{j}^{y}\left(t\right)\right) & = & \frac{{\theta_{j}^{1}}^{y}-\theta_{j}\left(t\right)}{{\theta_{j}^{1}}^{y}-{\theta_{j}^{2}}^{y}} \end{array}\right.$$

$${\bar{\mu }}_{j}^{1}\left(\theta_{j}^{y}\left(t\right)\right)+{\bar{\mu }}_{j}^{2}\left(\theta_{j}^{y}\left(t\right)\right)=1,\;\forall t$$

Replacing (6) and (8) in (3), we obtain:

(10) $$\left\{\begin{array}{c} B_{i}\left(t\right)=B_{i}+\sum_{j=1}^{n_{\theta_{u}}}\sum_{k=1}^{2}{\tilde{\mu }}_{j}^{k}\left(\theta_{j}\left(t\right)\right){\theta_{j}^{k}}^{u}{\bar{B}}_{ij}\\ C\left(t\right)=\left(I+\sum_{j=1}^{n_{\theta_{y}}}\sum_{k=1}^{2}{\bar{\mu }}_{j}^{k}\left(\theta_{j}^{y}\left(t\right)\right){\theta_{j}^{k}}^{y}F_{j}\right)C \end{array}\right.$$

#### 3.1.3. Unified Polytopic Representation of the CPS Under Data Deception Attacks

A key step in the proposed framework consists of transforming the attack-dependent system into a unified convex representation. Such a reformulation allows the effects of both actuator and sensor attacks to be embedded within a common set of scheduling variables, thereby providing a convenient basis for estimation, diagnosis, and resilient control design.

To this end, the convex-sum properties of the weighting functions associated with the attack parameters are exploited to derive a global polytopic model. More specifically, the weighting functions ${\tilde{\mu }}_{j}\left(\theta_{j}^{u}\left(t\right)\right)$ and ${\bar{\mu }}_{j}\left(\theta_{j}^{y}\left(t\right)\right)$, associated with actuator and sensor attacks, are combined to construct a common interpolation structure for all parameter-varying matrices. As a result, the attacked system can be represented exactly as a convex combination of linear submodels while preserving the original dependence on the malicious perturbations. Following the procedure reported in [23], Equation (10) can be rewritten as:

(11) $$\begin{array}{c} \left\{\begin{array}{ccc} B_{i}\left(t\right)\; & = & \;\sum_{j=1}^{n_{\theta_{u}}}\left[\left[\left({\tilde{\mu }}_{j}^{1}\left(\theta_{j}^{u}\left(t\right)\right){\theta_{j}^{1}}^{u}+{\tilde{\mu }}_{j}^{2}\left(\theta_{j}^{u}\left(t\right)\right){\theta_{j}^{2}}^{u}\right){\bar{B}}_{ij}\right]\right]\times \\ \; & \; & \left[\prod_{\begin{array}{c} k=1\\ k\neq j \end{array}}^{n_{\theta_{u}}}\sum_{m=1}^{2}{\tilde{\mu }}_{k}^{m}\left(\theta_{k}^{u}\left(t\right)\right)\right]+B_{i}\\ \; & = & B_{i}+\sum_{j=1}^{2^{n_{\theta_{u}}}}\tilde{\mu_{j}}\left(\theta^{u}\left(t\right)\right){\bar{B}}_{ij}\\ C(t) & = & \left(I+\sum_{j=1}^{2^{n_{\theta_{y}}}}\bar{\mu_{j}}\left(\theta^{y}\left(t\right)\right){\bar{F}}_{j}\right)C \end{array}\right. \end{array}$$

with

(12) $$\left\{\begin{array}{c} \tilde{\mu_{j}}\left(\theta^{u}\left(t\right)\right)=\prod_{k=1}^{n_{\theta_{u}}}{\tilde{\mu }}_{k}^{\sigma_{j}^{k}}\left(\theta_{k}^{u}\left(t\right)\right)\\ {\bar{B}}_{ij}=\sum_{k=1}^{n_{\theta_{u}}}{\theta_{k}^{u}}^{\sigma_{j}^{k}}{\bar{B}}_{ik} \end{array}\right.$$

and

(13) $$\left\{\begin{array}{c} \bar{\mu_{j}}\left(\theta^{y}\left(t\right)\right)=\prod_{k=1}^{n_{\theta_{y}}}{\bar{\mu }}_{k}^{\sigma_{j}^{k}}\left(\theta_{k}^{y}\left(t\right)\right)\\ {\bar{F}}_{j}=\sum_{k=1}^{n_{\theta_{y}}}{\theta_{k}^{y}}^{\sigma_{j}^{k}}F_{j} \end{array}\right.$$

where the global weighting functions $\tilde{\mu }j\left(\theta^{u}\left(t\right)\right)$ and $\bar{\mu }j\left(\theta^{y}\left(t\right)\right)$ satisfy the standard convexity conditions. These functions determine the contribution of each local model to the overall polytopic representation and ensure a smooth interpolation between the different operating regimes induced by the attack parameters.

The index $\sigma_{j}^{k}\in 1,2$ specifies the sector associated with the *k*-th scheduling parameter in the *j*-th vertex of the polytope. More precisely, it identifies which local weighting function, namely ${\tilde{\mu }}_{k}^{1}$ or ${\tilde{\mu }}_{k}^{2}$ (respectively ${\bar{\mu }}_{k}^{1}$ or ${\bar{\mu }}_{k}^{2}$), is selected in the construction of the *j*-th submodel. Consequently, each submodel corresponds to a unique combination of parameter-sector assignments, while the complete set of combinations defines the vertices of the polytopic representation. The correspondence between the submodel index *j* and the sector-selection indices $\sigma_{j}^{k}$ is established according to:

(14) $$j\;=\;2^{n_{\theta_{u}}-1}\sigma_{j}^{1}+2^{n_{\theta_{u}}-2}\sigma_{j}^{2}+\ldots +2^{0}\sigma_{j}^{n_{\theta_{u}}}-\left(2^{1}+2^{2}+\ldots +2^{n_{\theta_{u}}-1}\right)$$

for the actuator, and

(15) $$j\;=\;2^{n_{\theta_{y}}-1}\sigma_{j}^{1}+2^{n_{\theta_{y}}-2}\sigma_{j}^{2}+\ldots +2^{0}\sigma_{j}^{n_{\theta_{y}}}-\left(2^{1}+2^{2}+\ldots +2^{n_{\theta_{y}}-1}\right)$$

for the sensor.

Finally, using Equation (11), the nonlinear LPV system (2) becomes:

(16) $$\left\{\begin{array}{ccc} \dot{x}\left(t\right) & = & \sum_{i=1}^{r}\sum_{j=1}^{2^{n_{\theta_{u}}}}\mu_{i}\left(x\left(t\right)\right)\tilde{\mu_{j}}\left(\theta^{u}\left(t\right)\right)\left(A_{i}x\left(t\right)+B_{ij}u\left(t\right)\right)\\ y(t) & = & \sum_{k=1}^{2^{n_{\theta_{y}}}}\bar{\mu_{k}}\left(\theta^{y}\left(t\right)\right){\tilde{C}}_{k}x\left(t\right) \end{array}\right.$$

(17) $$\begin{array}{c} B_{ij}=B_{i}+{\bar{B}}_{ij}\\ {\tilde{C}}_{k}=C+{\bar{F}}_{k}C \end{array}$$

### 3.2. State and Cyber-Attack Estimation Under Uncertainty

From the polytopic representation derived in (16), a model-based observer is developed to simultaneously estimate the system states and the actuator/sensor data-deception attacks. The proposed estimation framework exploits the structure of the attacked system to reconstruct both the physical states and the malicious signals affecting the sensing and actuation channels. Since cyber-attacks can significantly degrade estimation accuracy and compromise monitoring performance, robustness with respect to attack-induced disturbances constitutes a key design requirement. To this end, an $L_{2}$-performance criterion is adopted, aiming to attenuate the influence of unknown attacks on the state- and attack-estimation errors. The resulting observer therefore combines state reconstruction capabilities with disturbance-rejection properties, providing a resilient estimation mechanism suitable for safety-critical cyber-physical systems.

The proposed joint state-and-attack observer is described by:

(18) $$\left\{\begin{array}{c} \dot{\hat{x}}\left(t\right)=\sum_{i=1}^{r}\sum_{j=1}^{2^{n_{\theta_{u}}}}\mu_{i}\left(\hat{x}\left(t\right)\right)\tilde{\mu_{j}}\left(\hat{\theta^{u}}\left(t\right)\right)\left(A_{i}x\left(t\right)+B_{ij}u\left(t\right)+L_{ij}\left(y\left(t\right)-\hat{y}\left(t\right)\right)\right)\\ \dot{\hat{\theta^{u}}}\left(t\right)=\sum_{i=1}^{r}\sum_{j=1}^{2^{n_{\theta_{u}}}}\mu_{i}\left(\hat{x}\left(t\right)\right)\tilde{\mu_{j}}\left(\hat{\theta^{u}}\left(t\right)\right)\left(K_{ij}^{u}\left(y\left(t\right)-\hat{y}\left(t\right)\right)-\alpha_{ij}^{u}{\hat{\theta }}^{u}\left(t\right)\right)\\ \dot{\hat{\theta^{y}}}\left(t\right)=\sum_{i=1}^{r}\sum_{k=1}^{2^{n_{\theta_{y}}}}\mu_{i}\left(\hat{x}\left(t\right)\right)\bar{\mu_{k}}\left(\hat{\theta^{y}}\left(t\right)\right)\left(K_{ik}^{y}\left(y\left(t\right)-\hat{y}\left(t\right)\right)-\alpha_{ik}^{y}\hat{\theta^{y}}\left(t\right)\right)\\ \hat{y}\left(t\right)=\sum_{k=1}^{2^{n_{\theta_{y}}}}\bar{\mu_{k}}\left(\hat{\theta^{y}}\left(t\right)\right){\tilde{C}}_{k}\hat{x}\left(t\right) \end{array}\right.$$

where $L_{ij}\in R^{\;n_{x}\times m}$, $K_{ij}^{u}\in R^{\;n\times m}$, $\alpha_{ij}^{u}\in R^{\;n\times n}$, $K_{ik}^{y}\in R^{\;m\times m}$ and $\alpha_{ik}^{y}\in R^{\;m\times m}$ denote the observer gain matrices. These gains are determined so as to guarantee the convergence of both the state- and attack-estimation errors while simultaneously satisfying the robustness and performance requirements introduced in the control design.

To evaluate the observer performance, the state-estimation error and the actuator/sensor attack-estimation errors are defined as:

(19) $$\begin{array}{cccc} e_{x}\left(t\right) & =x\left(t\right)-\hat{x}\left(t\right),\;e_{\theta^{u}}\left(t\right) & =\theta^{u}\left(t\right)-{\hat{\theta }}^{u}\left(t\right),\;e_{\theta^{y}}\left(t\right) & =\theta^{y}\left(t\right)-{\hat{\theta }}^{y}\left(t\right). \end{array}$$

The derivation of the error dynamics requires expressing the system equations in terms of the estimated scheduling variables. To this end, the convex-sum properties of the weighting functions are exploited following the methodology developed in [23]. Consequently, system (16) can be reformulated as

(20) $$\left\{\begin{array}{c} \dot{x}\left(t\right)=\sum_{i=1}^{r}\sum_{j=1}^{2^{n_{\theta_{u}}}}\left[\mu_{i}\left(\hat{x}\left(t\right)\right)\tilde{\mu_{j}}\left(\hat{\theta^{u}}\left(t\right)\right)\left(A_{i}x\left(t\right)+B_{ij}u\left(t\right)\right)+,\delta ij\left(t\right)\left(A_{i}x\left(t\right)+B_{ij}u\left(t\right)\right)\right]\\ y\left(t\right)=\sum_{k=1}^{2^{n_{\theta_{y}}}}\left[\bar{\mu_{k}}\left(\hat{\theta^{y}}\left(t\right)\right){\tilde{C}}_{k}x\left(t\right)+\bar{\delta_{k}}\left(t\right){\tilde{C}}_{k}x\left(t\right)\right] \end{array}\right.$$

where $\delta_{ij}\left(t\right)$ and $\bar{\delta_{k}}\left(t\right)$ denote the modeling error terms induced by the use of estimated weighting functions, defined as follows:

(21) $$\delta_{ij}\left(t\right)=\mu_{i}\left(x\left(t\right)\right)\tilde{\mu_{j}}\left(\theta^{u}\left(t\right)\right)-\mu_{i}\left(\hat{x}\left(t\right)\right)\tilde{\mu_{j}}\left(\hat{\theta^{u}}\left(t\right)\right)$$

(22) $$\bar{\delta_{k}}\left(t\right)=\bar{\mu_{k}}\left(\theta^{y}\left(t\right)\right)-\bar{\mu_{k}}\left(\hat{\theta^{y}}\left(t\right)\right)$$

From the convexity properties of the weighting functions, these terms satisfy the bounds:

(23) $$-1\leq \delta_{ij}\left(t\right)\leq 1,\;-1\leq \bar{\delta_{k}}\left(t\right)\leq 1.$$

The representation in (20) reformulates the system dynamics exclusively in terms of estimated variables weighting functions, namely $\mu_{i}\left(\hat{x}\left(t\right)\right)$, $\tilde{\mu_{j}}\left(\hat{\theta^{u}}\left(t\right)\right)$, and $\bar{\mu_{k}}\left(\hat{\theta^{y}}\left(t\right)\right)$, while the associated modeling error is captured through the bounded terms $\delta_{ij}\left(t\right)$ and $\bar{\delta_{k}}\left(t\right)$. This decomposition considerably facilitates the derivation of the state- and attack-estimation error dynamics and provides a suitable foundation for the following stability and performance analysis.

Let us define now:

(24) $$\Delta A\left(t\right)=\sum_{i=1}^{r}\sum_{j=1}^{2^{n_{\theta_{u}}}}\delta ij\left(t\right)A_{i}=A\Sigma \left(t\right)E_{A}$$

(25) $$\Delta B\left(t\right)=\sum_{i=1}^{r}\sum_{j=1}^{2^{n_{\theta_{u}}}}\delta_{ij}\left(t\right)B_{ij}=B\Sigma \left(t\right)E_{B}$$

(26) $$\Delta C\left(t\right)=\sum_{k=1}^{2^{n_{\theta_{y}}}}\bar{\delta_{k}}\left(t\right){\tilde{C}}_{k}=C\bar{\Sigma }\left(t\right)E_{C}$$

with

(27) $$A=\left[\begin{array}{ccc} \underset{2^{n_{\theta_{u}}times}}{\underset{︸}{\begin{array}{ccc} A_{1} & \ldots & A_{1} \end{array}}} & \ldots & \underset{2^{n_{\theta_{u}}times}}{\underset{︸}{\begin{array}{ccc} A_{r} & \ldots & A_{r} \end{array}}} \end{array}\right]$$

(28) $$B=\left[\begin{array}{ccc} B_{11} & \ldots & B_{r2^{n}} \end{array}\right]$$

(29) $$C=\left[\begin{array}{ccc} {\tilde{C}}_{1} & \ldots & {\tilde{C}}_{2^{n_{\theta_{y}}}} \end{array}\right]$$

(30) $$\begin{array}{c} \Sigma \left(t\right)=\mathrm{diag}(\delta_{11}\left(t\right),\ldots ,\delta_{r2^{n}}\left(t\right)),\\ \bar{\Sigma }\left(t\right)=\mathrm{diag}\left(\bar{\delta_{1}}\left(t\right),\ldots ,\bar{\delta_{2^{n_{\theta_{y}}}}}\left(t\right)\right) \end{array}$$

(31) $$\begin{array}{c} E_{A}={\left[\begin{array}{ccc} I_{n_{x}} & \ldots & I_{n_{x}} \end{array}\right]}^{T},\;E_{B}={\left[\begin{array}{ccc} I_{n_{u}} & \ldots & I_{n_{u}} \end{array}\right]}^{T}\\ E_{C}={\left[\begin{array}{ccc} I_{2^{n_{\theta_{y}}}} & \ldots & I_{2^{n_{\theta_{y}}}} \end{array}\right]}^{T}={\left[\begin{array}{ccc} I_{2^{m}} & \ldots & I_{2^{m}} \end{array}\right]}^{T} \end{array}$$

Thanks to (23) and the definitions in (30), we have:

(32) $$\Sigma^{T}\left(t\right)\Sigma \left(t\right)\leq I,\;{\bar{\Sigma }}^{T}\left(t\right)\bar{\Sigma }\left(t\right)\leq I$$

Using the above definitions (24)–(31), system (20) is then written as an uncertain system given by:

(33) $$\left\{\begin{array}{c} \dot{x}\left(t\right)=\sum_{i=1}^{r}\sum_{j=1}^{2^{n_{\theta_{u}}}}\mu_{i}\left(\hat{x}\left(t\right)\right)\tilde{\mu_{j}}\left(\hat{\theta^{u}}\left(t\right)\right)\left(\left(A_{i}+\Delta A\left(t\right)\right)x\left(t\right)+\left(B_{ij}+\Delta B\left(t\right)\right)u\left(t\right)\right)\\ y\left(t\right)=\sum_{k=1}^{2^{n_{\theta_{y}}}}\bar{\mu_{k}}\left(\hat{\theta^{y}}\left(t\right)\right)\left({\tilde{C}}_{k}+\Delta C\left(t\right)\right)x\left(t\right) \end{array}\right.$$

In the sequel, building upon the system representations (16) and (33), the control problem for cyber-physical systems under cyber-attacks is addressed. An observer-based framework is considered, where a state observer reconstructs the system state and a controller generates the control input using estimated information.

The objective is to develop resilient control strategies that guarantee closed-loop stability and performance despite malicious attacks affecting sensing and actuation channels. In parallel, the proposed approach enables joint estimation of system states and attack signals, thereby providing detection and isolation capabilities within a unified estimation–control architecture. The observer and controller gains are derived using a Lyapunov-based stability analysis combined with Linear Matrix Inequalities (LMIs), leading to convex sufficient conditions ensuring stability and performance guarantees.

In addition, an event-triggered observer–controller co-design is proposed for nonlinear cyber-physical systems. The scheme explicitly accounts for network-induced constraints such as limited communication resources and bandwidth limitations, which are embedded into the closed-loop dynamics. The resulting co-design jointly synthesizes a state-and-attack observer and an event-triggered controller. Stability of the resulting augmented system is established using Lyapunov arguments under the proposed event-triggered implementation.

## 4. Robust Output $H_{\infty }$ T-S Control

Fault diagnosis, fault-tolerant control, and resilient estimation constitute a classical yet still highly active research problem in control engineering. Early works such as [27,28] addressed fault detection and isolation by analyzing residual signals obtained from the discrepancy between measured outputs and model-based predictions. In contrast to these approaches, the objective of the present work is not limited to detection, but extends to the explicit estimation of attack signals, which is essential for the synthesis of robust and attack-tolerant control laws. Indeed, the absence of reliable detection and estimation mechanisms may lead to severe degradation of system performance and potential physical damage to the plant.

In this section, we focus on integrity attacks, commonly referred to as deception attacks or false-data injection attacks, which directly compromise sensor and actuator information. To counter such malicious interventions, various detection schemes have been proposed in the literature [29,30]. The approach adopted in this work builds upon the framework developed in [25], aiming at the simultaneous and exact reconstruction of both system states and time-varying attack signals. We consider that the attacker injects false information into the control loop. These effects are modeled as multiplicative time-varying faults acting on the sensing and actuation channels. The polytopic Takagi–Sugeno (T-S) framework is then employed to reconstruct these signals in real time, enabling a unified estimation and control design.

The main objective is to design T-S observer and controller gains such that the following properties are satisfied:

- The closed-loop system (33) remains asymptotically stable in the presence of deception attacks.
- The effect of external disturbances, including actuator attacks, is attenuated in the $H_{\infty }$ sense. More precisely, for a given scalar γ>0, an observer of the form (18) and a PDC controller (34) are designed such that the attenuation condition (41) holds. The corresponding synthesis conditions are provided in Lemma 2.

Finally, we consider the nonlinear system subject to data deception attacks described by (2), together with the polytopic T-S observer (18) for the estimation of unmeasurable states and unknown time-varying parameters corresponding to actuator and sensor attacks. The associated PDC (Parallel Distributed Compensation) controller is defined by:

(34) $$u\left(t\right)=-\sum_{l=1}^{r}h_{l}\left(\hat{x}\left(t\right)\right)\Omega_{l}\hat{x}\left(t\right).$$

The estimation error dynamics are derived from (33) and (19) as follows:

(35) $$\left\{\begin{array}{c} {\dot{e}}_{x}\left(t\right)=\sum_{i=1}^{r}\sum_{j=1}^{2^{n_{\theta_{u}}}}\sum_{k=1}^{2^{n_{\theta_{y}}}}\sum_{l=1}^{r}\mu_{i}\left(\hat{x}\left(t\right)\right)\tilde{\mu_{j}}\left(\hat{\theta^{u}}\left(t\right)\right)\bar{\mu_{k}}\left(\hat{\theta^{y}}\left(t\right)\right)\mu_{l}\left(\hat{x}\left(t\right)\right)(\left(A_{i}-L_{ij}{\tilde{C}}_{k}+\Delta B\left(t\right)\Omega_{l}\right)e_{x}\left(t\right)\\ \;\;\;+(\Delta A\left(t\right)-\Delta B\left(t\right)\Omega_{l}-L_{ij}\Delta C\left(t\right))x\left(t\right))\\ {\dot{e}}_{\theta^{u}}\left(t\right)=\sum_{i=1}^{r}\sum_{j=1}^{2^{n_{\theta_{u}}}}\sum_{k=1}^{2^{n_{\theta_{y}}}}\mu_{i}\left(\hat{x}\left(t\right)\right)\tilde{\mu_{j}}\left(\hat{\theta^{u}}\left(t\right)\right)\bar{\mu_{k}}\left(\hat{\theta^{y}}\left(t\right)\right)(-K_{ij}^{u}{\tilde{C}}_{k}e_{x}\left(t\right)-\alpha_{ij}^{u}e_{\theta^{u}}\left(t\right)-K_{ij}^{u}\Delta C\left(t\right)x\left(t\right)\\ \;\;\;\;\;+\alpha_{ij}^{u}\theta^{u}\left(t\right)+\dot{\theta^{u}}\left(t\right))\\ {\dot{e}}_{\theta^{y}}\left(t\right)=\sum_{i=1}^{r}\sum_{k=1}^{2^{n_{\theta_{y}}}}\mu_{i}\left(\hat{x}\left(t\right)\right)\bar{\mu_{k}}\left(\hat{\theta^{y}}\left(t\right)\right)\left(-K_{ik}^{y}{\tilde{C}}_{k}e_{x}\left(t\right)-\alpha_{ik}^{y}e_{\theta^{y}}\left(t\right)-K_{ik}^{y}\Delta C\left(t\right)x\left(t\right)+\alpha_{ik}^{y}\theta^{y}\left(t\right)+\dot{\theta^{y}}\left(t\right)\right) \end{array}\right.$$

Combining the system model (33), the observer (18), the PDC controller, and the estimation error definitions (35), the resulting closed-loop system can be expressed as follows:

(36) $$\begin{array}{c} {\dot{x}}_{a}\left(t\right)=\sum_{i=1}^{r}\sum_{j=1}^{2^{n_{\theta_{u}}}}\sum_{k=1}^{2^{n_{\theta_{y}}}}\sum_{l=1}^{r}\mu_{i}\left(\hat{x}\left(t\right)\right)\tilde{\mu_{j}}\left(\hat{\theta^{u}}\left(t\right)\right)\bar{\mu_{k}}\left(\hat{\theta^{y}}\left(t\right)\right)\mu_{l}\left(\hat{x}\left(t\right)\right)\left(\Phi_{ijkl}x_{a}\left(t\right)+\Psi_{ijk}\omega \left(t\right)\right) \end{array}$$

The augmented state vector is defined as $x_{a}\left(t\right)={\left(\begin{array}{cccc} x(t) & e_{x}\left(t\right) & e_{\theta }^{u}\left(t\right) & e_{\theta }^{y}\left(t\right) \end{array}\right)}^{T}$ which consists of the system state, the state-estimation error, and the attack-estimation errors associated with actuator and sensor channels.

The vector $\omega \left(t\right)={\left(\begin{array}{cccc} \theta^{u}\left(t\right) & {\dot{\theta }}^{u}\left(t\right) & \theta^{y}\left(t\right) & {\dot{\theta }}^{y}\left(t\right) \end{array}\right)}^{T}$ represents the exogenous input, composed of the attack signals and their time derivatives, which are assumed to be unknown but bounded.

Matrices $\Phi_{ijkl}$ and $\Psi_{ijk}$ are defined as follows:

(37) $$\Phi_{ijk}=\left(\begin{array}{cccc} \Phi_{ijkl}^{1} & \left(B_{ij}+\Delta B\left(t\right)\right)\Omega_{l} & 0 & 0\\ \Delta A\left(t\right)-\Delta B\left(t\right)\Omega_{l}-L_{ij}\Delta C\left(t\right) & A_{i}-L_{ij}{\tilde{C}}_{k}+\Delta B\left(t\right)\Omega_{l} & -K_{ij}^{u}{\tilde{C}}_{k} & 0\\ -K_{ij}^{u}\Delta C\left(t\right) & -K_{ij}C & -\alpha_{ij}^{u} & 0\\ -K_{ik}^{y}\Delta C\left(t\right) & -K_{ik}^{y}{\tilde{C}}_{k} & 0 & -\alpha_{ik}^{y} \end{array}\right)$$

with $\Phi_{ijkl}^{1}=A_{i}-B_{ij}\Omega_{l}+\Delta A\left(t\right)-\Delta B\left(t\right)\Omega_{l}$ and

(38) $$\Psi_{ijk}=\left(\begin{array}{cccc} 0 & 0 & 0 & 0\\ 0 & 0 & 0 & 0\\ \alpha_{ij}^{u} & I & 0 & 0\\ 0 & 0 & \alpha_{ik}^{y} & I \end{array}\right)$$

Combining (36) with the nominal attack-free output $y_{n}\left(t\right)=Cx\left(t\right)$, the closed-loop system can be expressed as follows:

(39) $$\begin{array}{c} \left(\begin{array}{c} {\dot{x}}_{a}\left(t\right)\\ y_{n}\left(t\right) \end{array}\right)=\sum_{i=1}^{r}\sum_{j=1}^{2^{n_{\theta_{u}}}}\sum_{k=1}^{2^{n_{\theta_{y}}}}\sum_{l=1}^{r}\mu_{i}\left(\hat{x}\left(t\right)\right)\tilde{\mu_{j}}\left(\hat{\theta^{u}}\left(t\right)\right)\bar{\mu_{k}}\left(\hat{\theta^{y}}\left(t\right)\right)\mu_{l}\left(\hat{x}\left(t\right)\right)\left(\begin{array}{cc} \Phi_{ijkl} & \Psi_{ijk}\\ \bar{C} & 0 \end{array}\right)\left(\begin{array}{c} x_{a}\left(t\right)\\ \omega (t) \end{array}\right) \end{array}$$

s.t.

(40) $$y_{n}\left(t\right)=Cx\left(t\right)=\left(\begin{array}{cccc} C & 0 & 0 & 0 \end{array}\right)x_{a}\left(t\right)=\bar{C}x_{a}\left(t\right)$$

Before introducing the stabilization and control results, the following definition and lemma are recalled:

**Definition** **1.** *Given a positive scalar γ>0, the system (39) is said to achieve an $H_{\infty }$ performance level γ if it is exponentially stable and satisfies the following disturbance attenuation condition:*

(41) $$\int_{0}^{\infty }\left(y_{n}^{T}\left(t\right)\;y_{n}\left(t\right)-\gamma^{2}\;\omega^{T}\left(t\right)\omega \left(t\right)\right)dt<0,$$

*where γ denotes the prescribed level of disturbance attenuation.*

**Lemma** **1.** *Based on Lyapunov stability theory, the continuous-time system (19) is $H_{\infty }$ stable with attenuation level γ if there exists a positive definite symmetric matrix $P=P^{T}>0$ such that*

(42) $$\left[\begin{array}{ccc} \Phi_{ijkl}^{T}P+P\Phi_{ijkl} & P\Psi_{ijk} & {\bar{C}}^{T}\\ {}* & -\gamma^{2}I & 0\\ {}* & * & -I \end{array}\right]<0,$$

*for all i,l=1,…,r, $j=1,\ldots ,2^{n_{\theta_{u}}}$, and $k=1,\ldots ,2^{n_{\theta_{y}}}$.*

To reduce the conservatism of the stability conditions stated in Lemma 1, the following alternative formulation is introduced, following [31,32].

**Lemma** **2** ([33])**.** *Let γ>0 be a given scalar. Consider the uncertain polytopic Takagi–Sugeno system (33) in a closed loop with the PDC controller (34). Assume that there exist matrices P and $Z_{ijkl}$ such that $P=P^{T}>0$, and $Z_{ijkl}$ satisfy the symmetry properties $Z_{ijkl}=Z_{ijkl}^{T}$ for i=k and $Z_{ljki}=Z_{ijkl}^{T}$ for i≠k, with i,l=1,…,r, $j=1,\ldots ,2^{n_{\theta_{u}}}$, and $k=1,\ldots ,2^{n_{\theta_{y}}}$, and such that the set of matrix inequalities (43)–(45) is satisfied. Then, the closed-loop system (39) is asymptotically stable and achieves an $H_{\infty }$ performance level γ, i.e., the $H_{\infty }$ norm of the corresponding polytopic closed-loop system is strictly bounded by γ.*

(43) $$\left[\begin{array}{cc} \Phi_{ijki}^{T}P+P\Phi_{ijki} & P\Psi_{ijk}\\ {}* & -\gamma^{2}I \end{array}\right]<Z_{ijki}$$

$$i,l=1,\ldots ,r,j=1,\ldots ,2^{n_{\theta_{u}}},k=1,\ldots ,2^{n_{\theta_{y}}}$$

(44) $$\left[\begin{array}{cc} {\left(*\right)}^{T}P+P\left(\Phi_{ijkl}+\Phi_{ljki}\right) & 2P\Psi_{ijk}\\ {}* & 2\Psi_{ijk}^{T}P \end{array}\right]<Z_{ijkl}+Z_{ljki}$$

$$i\neq l,j=1,\ldots ,2^{n_{\theta_{u}}},k=1,\ldots ,2^{n_{\theta_{y}}}$$

(45) $${\left[\begin{array}{cccc} Z_{1jk1} & \ldots & Z_{1jkr} & {\bar{C}}^{T}\\ \vdots & \ddots & \vdots & \vdots \\ Z_{rjk1} & \ldots & Z_{rjkr} & {\bar{C}}^{T}\\ \bar{C} & \ldots & \bar{C} & -I \end{array}\right]}_{j=1,\ldots ,2^{n_{\theta_{u}}},k=1,\ldots ,2^{n_{\theta_{y}}}}<0$$

By replacing $\Phi_{ijkl}$ and $\Psi_{ijk}$ with their expressions, with some change of variables and a classical linearization procedure (Schur’s complement and bounded real lemma), and the following Lemma 3 (the idea is to bound the time-varying terms):

**Lemma** **3** ([28])**.** *Consider two matrices X and Y with appropriate dimensions, a time-varying matrices Δ(t) and a positive scalar ε. The following property is verified*

(46) $$X^{T}\Delta^{T}\left(t\right)Y+Y^{T}\Delta \left(t\right)X\leq \varepsilon X^{T}X+\varepsilon^{-1}Y^{T}Y$$

*for $\Delta^{T}\left(t\right)\Delta \left(t\right)\leq I$.*

The resulting constraints can be efficiently solved using standard convex optimization tools, or alternatively by means of dedicated solvers for bilinear matrix inequalities, such as the PenBMI MATLAB -R2020b toolbox (see [34,35] for representative examples).

An important advantage of the proposed approach lies in its unified synthesis framework, which enables the simultaneous design of both controller and observer gains within a single-step procedure. This contrasts with classical two-step design methodologies, such as those reported in [36], where observer and controller synthesis are typically performed sequentially, potentially leading to increased conservatism.

### Calculation Details

In the following, a detailed computational procedure is provided for solving the matrix inequalities (42)–(44). For simplicity of presentation, we consider the case where the system is affected by a single actuator attack, i.e., $n_{\theta_{y}}=1$ and y(t)=Cx(t).

**Remark** **2.** *It is important to highlight that although the number of vertices increases with the number of scheduling variables and attack channels, this growth primarily affects the offline synthesis stage, during which the observer and controller gains are computed. The online implementation only requires the evaluation of the scheduling functions and the interpolation of precomputed gains, resulting in a significantly lower computational burden suitable for real-time execution. Furthermore, the practical feasibility of the polytopic framework has previously been demonstrated on a real biological wastewater treatment plant involving a substantially larger number of submodels, confirming the applicability of the proposed methodology beyond the illustrative example considered in this paper. Nevertheless, further investigations on scalability for very large-scale networked CPSs remain an important perspective for future work.*

It is important to highlight that this assumption is made exclusively for presentation purposes and that the proposed framework, without loss of generality, naturally extends to the general case of multiple simultaneous actuator attacks by appropriately augmenting the attack vector and the associated system matrices.

Under these assumptions, the resulting uncertain system with bounded external disturbances can be written as follows:

(47) $${\dot{x}}_{a}\left(t\right)=\sum_{i=1}^{r}\sum_{j=1}^{2}\sum_{k=1}^{r}h_{i}\left(\hat{x}\right)\mu_{j}\left(\hat{a^{u}}\right)h_{k}\left(\hat{x}\right)\left(\Phi_{ijk}x_{a}\left(t\right)+\Psi_{ij}\omega \left(t\right)\right)$$

s.t. $x_{a}\left(t\right)={\left(\begin{array}{ccc} x(t) & e_{x}\left(t\right) & e_{a^{u}}\left(t\right) \end{array}\right)}^{T}$ and $\omega \left(t\right)={\left(\begin{array}{cc} a^{u}\left(t\right) & \dot{a^{u}}\left(t\right) \end{array}\right)}^{T}$.

Matrices $\Phi_{ijk}$ and $\Psi_{ij}$ are defined as follows:

(48) $$\Phi_{ijk}=\left(\begin{array}{ccc} \Phi_{ijk}^{1} & \left(B_{ij}+\Delta B\left(t\right)\right)\Omega_{k} & 0\\ \Delta A\left(t\right)-\Delta B\left(t\right)\Omega_{k} & A_{i}-L_{ij}C & 0\\ 0 & -K_{ij}C & -\alpha_{ij}^{u} \end{array}\right)$$

with $\Phi_{ijk}^{1}=A_{i}-B_{ij}\Omega_{k}+\Delta A\left(t\right)-\Delta B\left(t\right)\Omega_{k}$ and

(49) $$\Psi_{ij}=\left(\begin{array}{cc} 0 & 0\\ 0 & 0\\ \alpha_{ij}^{u} & I \end{array}\right)$$

Let us now detail the stability condition given by Lemma 1 for this system, i.e.,

(50) $${\left[\begin{array}{ccc} \Phi_{ijk}^{T}P+P\Phi_{ijk} & P\Psi_{ij} & {\bar{C}}^{T}\\ {}* & -\gamma^{2}I & 0\\ {}* & * & -I \end{array}\right]}_{i,k=1,\ldots ,r,j=1,2}<0$$

In order to handle this constraint, each term is first explicitly written using its analytical expression. The resulting formulation is then decomposed into constant and time-varying components. The time-varying terms, due to the convex sum property of the weighting functions, are appropriately bounded, which enables the use of standard convex optimization and bilinear matrix inequality solvers as discussed previously. Based on definitions (48) and (49), and by considering a diagonal structure for the matrix *P*, (i.e., $P=\left(\begin{array}{ccc} P_{1} & 0 & 0\\ 0 & P_{2} & 0\\ 0 & 0 & P_{3} \end{array}\right)$, where $P_{1}$, $P_{2}$ and $P_{3}$ are positive symmetric matrix), the constraint (50) is rewritten as:

(51) $$Q_{ijk}+Q_{k}\left(t\right)+Q_{k}^{T}\left(t\right)<0,\;i,k=1,\ldots ,r,j=1,2$$

where $Q_{ijk}$ is given by:

(52) $$Q_{ijk}=\left(\begin{array}{cccccc} Q_{ijk}^{1} & P_{1}B_{ij}\Omega_{k} & 0 & 0 & 0 & C\\ {}* & Q_{ij}^{2} & -C^{T}K_{ij}^{T}P_{3} & 0 & 0 & 0\\ {}* & * & -2\alpha_{ij}P_{3} & P_{3}\alpha_{ij} & P_{3} & 0\\ {}* & * & * & -\gamma^{2}I & 0 & 0\\ {}* & * & * & * & -\gamma^{2}I & 0\\ {}* & * & * & * & * & -I \end{array}\right)$$

with $Q_{ijk}^{1}=P_{1}A_{i}-P_{1}B_{ij}\Omega_{k}+A_{i}^{T}P_{1}-\Omega_{k}^{T}B_{ij}^{T}P_{1}$ and $Q_{ij}^{2}=P_{2}A_{i}-P_{2}L_{ij}C+A_{2}^{T}P_{2}-C^{T}L_{ij}^{T}P_{2}$ Based on (24)–(26), $Q_{k}\left(t\right)$ is rewritten as:

(53) $$\begin{array}{c} Q_{k}\left(t\right)={\left(\begin{array}{cccccc} A^{T}P_{1} & A^{T}P_{2} & 0 & 0 & 0 & 0 \end{array}\right)}^{T}\Sigma \left(t\right)\left(\begin{array}{cccccc} E_{A} & 0 & 0 & 0 & 0 & 0 \end{array}\right)\\ +{\left(\begin{array}{cccccc} B^{T}P_{1} & 0 & 0 & 0 & 0 & 0 \end{array}\right)}^{T}\Sigma \left(t\right)\left(\begin{array}{cccccc} -E_{B}\Sigma_{k} & -E_{B}\Sigma_{k} & 0 & 0 & 0 & 0 \end{array}\right)\\ +{\left(\begin{array}{cccccc} 0 & B^{T}P_{2} & 0 & 0 & 0 & 0 \end{array}\right)}^{T}\Sigma \left(t\right)\left(\begin{array}{cccccc} -E_{B}\Sigma_{k} & 0 & 0 & 0 & 0 & 0 \end{array}\right) \end{array}$$

Based on property (32) and Lemma 3, $Q_{k}\left(t\right)+Q_{k}^{T}\left(t\right)$ is bounded as the following:

(54) $$Q_{k}\left(t\right)+Q_{k}^{T}\left(t\right)<\left(\begin{array}{cccccc} Q_{1} & Q_{3} & 0 & 0 & 0 & 0\\ Q_{2} & 0 & 0 & 0 & 0 & 0\\ 0 & 0 & 0 & 0 & 0 & 0\\ 0 & 0 & 0 & 0 & 0 & 0\\ 0 & 0 & 0 & 0 & 0 & 0\\ 0 & 0 & 0 & 0 & 0 & 0 \end{array}\right)$$

with $Q_{1}=\varepsilon_{A1}^{-1}P_{1}AA^{T}P_{1}+\varepsilon_{A1}E_{A}^{T}E_{A}+\varepsilon_{B1}^{-1}P_{1}BB^{T}P_{1}+\varepsilon_{B1}\Omega_{k}^{T}E_{B}^{T}E_{B}\Omega_{k}$$+\varepsilon_{A2}E_{A}^{T}E_{A}+$$\varepsilon_{B1}^{-1}P_{1}BB^{T}P_{1}+\varepsilon_{B2}\Omega_{k}^{T}E_{B}^{T}E_{B}\Omega_{k}$, $Q_{2}=\varepsilon_{A2}^{-1}P_{2}AA^{T}P_{2}+\varepsilon_{B2}^{-1}P_{2}BB^{T}P_{2}$, and $Q_{3}=\varepsilon_{B1}\Omega_{k}^{T}E_{B}^{T}E_{B}\Omega_{k}$.

Now, applying Schur’s complement with adequate change of variables, constraints (42)–(44) will be easily solved using the tools (PenBMI) presented above.

Although the $H_{\infty }$ control framework effectively addresses robustness and security concerns arising from disturbances, uncertainties, and data-deception attacks, these guarantees are obtained within a conventional periodic control framework, where measurements, communications, and control updates are executed at predetermined sampling instants regardless of the system’s actual behavior. While such an approach is well established, it may lead to excessive communication traffic, unnecessary control updates, and increased computational burden. These issues become particularly critical in modern cyber-physical and networked control systems, where communication bandwidth, processing capabilities, and energy resources are often limited. To address these challenges, attention has increasingly shifted toward event-triggered control strategies, in which information exchange and controller updates are driven by the system evolution rather than by a fixed time schedule. By transmitting data only when a prescribed triggering condition is satisfied, event-triggered schemes significantly reduce resource utilization while preserving the desired stability, robustness, and performance properties. Motivated by these considerations, the next section investigates the design of an event-triggered control strategy within the proposed polytopic $H_{\infty }$ framework, with the objective of maintaining robustness against uncertainties and cyber-attacks while reducing communication and computation requirements.

## 5. Polytopic Modeling of the Event-Triggered Control

The modeling framework adopted in this section builds upon previous results published in [22,23]. To reduce communication and computational burden, an event-triggered control strategy is introduced, in which the controller is updated only when predefined triggering conditions are satisfied. This leads to a joint control and co-scheduling architecture, where computation and communication resources are efficiently managed. One can refer to contributions like [37,38,39,40,41] for more details about the event-based observer–controller approaches.

Overall, the main contribution of this section is the development of an event-triggered observer–controller co-design framework for nonlinear cyber-physical systems. Network-induced constraints, such as limited communication resources, are explicitly incorporated into the system dynamics. The proposed co-design strategy enables the synthesis of a state-and-attack observer together with an event-triggered controller, ensuring stability of the resulting augmented system. Exploiting the sector nonlinearity representation together with Lyapunov stability theory, sufficient conditions are derived in terms of Bilinear Matrix Inequalities (BMIs), guaranteeing uniform and ultimate boundedness of the closed-loop signals. In addition, the estimation error is shown to converge to an origin-centered bounded set, while an $L_{2}$ performance index is achieved with respect to data-deception disturbances.

This work extends previous results obtained in [22,23] by incorporating event-triggered implementation within the polytopic framework. In the event-triggered control strategy, the control input is held constant between two successive triggering instants and updated only when the triggering condition is violated, following a classical sample-and-hold implementation. The observer-based state feedback controller under event-triggered transmission is formalized as follows [42]:

(55) $$u\left(t\right)=u\left(t_{k}\right)\;\forall t\in [t_{k},t_{k+1}[,k\in N$$

where the sequence ${\left\{t_{k}\right\}}_{k\in N}$ denotes the sequence of control update instants.

The next triggering instant $t_{k+1}$ is defined as

(56) $$t_{k+1}=\min\left\{t\geq t_{k}+T\;|\;f\left(\delta \left(t\right),\eta \left(t\right)\right)\geq 0\right\},$$

where T>0 is a minimum inter-event time ensuring the absence of Zeno behavior (i.e., an infinite number of updates happen in a finite time interval). The triggering function f(·) is designed based on Lyapunov stability arguments to guarantee an $L_{2}$ attenuation property from δ(t) to η(t), where

$$\eta \left(t\right)=\left[\begin{array}{c} e(t)\\ x(t) \end{array}\right],\;e\left(t\right)=x\left(t\right)-\hat{x}\left(t\right).$$

The signal δ(t) is defined as in [43,44,45]:

(57) $$\delta \left(t\right)=u\left(t_{k}\right)-u\left(t\right),$$

and quantifies the deviation between the sampled control input and its continuous-time counterpart.

The event-triggered control law is expressed component-wise as:

(58) $$u_{j}\left(t\right)=\left\{\begin{array}{c} u_{j}\left(t\right)\;\mathrm{if}\;\delta_{j}\left(t\right)<-\gamma_{j}\\ u_{j}\left(t_{k}\right)\;\mathrm{if}\;-\gamma_{j}<\delta_{j}\left(t\right)<\gamma_{j}\\ u_{j}\left(t\right)\;\mathrm{if}\;\delta_{j}\left(t\right)>\gamma_{j} \end{array}\right.$$

for $j=1,\ldots ,n_{u}$. $\delta_{j}\left(t\right)$ is defined as $\delta_{j}\left(t\right)=u_{j}\left(t_{k}\right)-u_{j}\left(t\right))$ and $\gamma_{j}$ is the considered threshold.

(58) can also be rewritten as:

(59) $$u_{j}\left(t\right)=\sum_{i=1}^{3}\mu_{i}^{j}\left(t\right)\left(\lambda_{i}^{j}u\left(t\right)+\left(1-\lambda_{i}^{j}\right)u\left(t_{k}\right)\right)$$

with the activation functions $\mu_{i}^{j}\left(t\right)$ defined as:

(60) $$\left\{\begin{array}{c} \mu_{1}^{j}\left(t\right)=\frac{1-\mathrm{sign}(\delta_{j}\left(t\right)+\gamma_{j})}{2}\\ \mu_{2}^{j}\left(t\right)=\frac{\mathrm{sign}\left(\delta_{j}\left(t\right)+\gamma_{j}\right)-sign\left(\delta_{j}\left(t\right)-\gamma_{j}\right)}{2}\\ \mu_{3}^{j}\left(t\right)=\frac{1+\mathrm{sign}(\delta_{j}\left(t\right)-\gamma_{j})}{2} \end{array}\right.$$

and the sign function given by:

(61) $$\mathrm{sign}\left(x\right):=\left\{\begin{array}{ccc} -1 & \mathrm{if} & x<0\\ 0 & \mathrm{if} & x=0\\ 1 & \mathrm{if} & x>0 \end{array}\right.$$

and the $\lambda_{i}$ values for i=1,2,3 are set to: $\lambda_{1}^{j}=1$, $\lambda_{2}^{j}=0$, $\lambda_{3}^{j}=1$.

By construction, the weighting functions associated with the polytopic event-triggered control law (59) satisfy the convex-sum property. Consequently,

(62) $$\sum_{i=1}^{3}\mu_{i}^{j}\left(t\right)=1,\;\forall t$$

**Remark** **3.** *The activation functions defined in (60) are discontinuous because of the use of the sign function. Therefore, the event-triggered control law can be interpreted as a piecewise-defined, or switching, polytopic control law. This discontinuity only affects the selection of the active region associated with the event-triggering condition. It does not require differentiating the activation functions in the Lyapunov analysis.*

*Indeed, the Lyapunov function considered in the sequel is common to all admissible sub-models. The derivative $\dot{V}\left(t\right)$ is computed along the trajectories of each active sub-model, and the convex-sum property of the weighting functions is then used to combine the corresponding inequalities. Hence, no term involving ${\dot{\mu }}_{i}^{j}\left(t\right)$, nor any derivative of the sign function, appears in the stability proof.*

*At switching instants, the control input may be discontinuous, but the system state remains continuous since no impulsive dynamics are introduced. Consequently, the Lyapunov function remains continuous, and its decrease can be interpreted in the piecewise-continuous sense. Since the same Lyapunov function guarantees the decrease condition for all admissible sub-models, the discontinuity induced by the sign-based activation functions does not compromise the validity of the Lyapunov stability proof.*

*It should be noted that the following stability conditions are derived using a common quadratic Lyapunov function for all sub-models generated by the sign-based event-triggered decomposition. Therefore, the proof does not rely on the differentiability of the activation functions, but only on their positivity and convex-sum property.*

**Remark** **4.** *To guarantee the applicability of the Lyapunov-based stability analysis, the convex-sum property must be preserved at the level of the overall control input rather than only for each individual component $u_{j}\left(t\right)$. Accordingly, each control component can be expressed as*

(63) $$\begin{array}{c} u_{j}\left(t\right)=\sum_{i=1}^{3}\mu_{i}^{j}\left(t\right)\left(\lambda_{i}^{j}u\left(t\right)+\left(1-\lambda_{i}^{j}\right)u\left(t_{k}\right)\right)\\ \;\;\;\;\times \left(\prod_{\begin{array}{c} k=1\\ k\neq j \end{array}}^{n_{u}}\sum_{l=1}^{3}\mu_{l}^{k}\left(t\right)\right). \end{array}$$

*By exploiting the convex-sum property of the weighting functions, the resulting event-triggered control law can be rewritten in the following compact polytopic form:*

(64) $$u\left(t\right)=\sum_{i=1}^{3^{n_{u}}}\mu_{i}\left(t\right)\left(\Lambda_{i}u\left(t\right)+\left(I_{n_{u}}-\Lambda_{i}\right)u\left(t_{k}\right)\right),$$

*which yields a polytopic representation composed of $3^{n_{u}}$ submodels. The global weighting functions $\mu_{i}\left(t\right)$ and the corresponding $\Lambda_{i}$ matrices are defined as*

(65) $$\left\{\begin{array}{ccc} \mu_{i}\left(t\right)\; & = & \prod_{j=1}^{n_{u}}\mu_{\sigma_{i}^{j}}^{j}\left(t\right),\\ \Lambda_{i}\; & = & \mathrm{diag}\;\left(\lambda_{\sigma_{i}^{1}}^{1},\ldots ,\lambda_{\sigma_{i}^{n_{u}}}^{n_{u}}\right). \end{array}\right.$$

*The indices $\sigma_{i}^{j}\in \left\{1,2,3\right\}$ identify the partition of the jth input involved in the ith submodel. The relation between the submodel index i and the indices $\sigma_{i}^{j}$ is given by*

$$i=3^{n_{u}-1}\sigma_{i}^{1}+3^{n_{u}-2}\sigma_{i}^{2}+\cdots +3^{0}\sigma_{i}^{n_{u}}-\sum_{k=1}^{n_{u}-1}3^{k}.$$

*Equivalently, the tuple $\left(\sigma_{i}^{1}-1,\ldots ,\sigma_{i}^{n_{u}}-1\right)$ corresponds to the base-3 representation of the integer i−1.*

### Polytopic Representation for Event-Triggered Observer–Controller Co-Design

The nonlinear system under consideration is described by the following polytopic representation:

(66) $$\left\{\begin{array}{ccc} \dot{x}\left(t\right) & = & \sum_{i=1}^{r}\mu_{i}\left(x\left(t\right)\right)\left(A_{i}x\left(t\right)+B_{i}\left(t\right)u\left(t\right)\right)\\ y(t) & = & C(t)x(t) \end{array}\right.$$

with $B_{i}\left(t\right)$ and C(t) given by:

(67) $$\left\{\begin{array}{c} B_{i}\left(t\right)=B_{i}+\sum_{j=1}^{n_{\theta_{u}}}\theta_{j}^{u}\left(t\right){\bar{B}}_{ij}\\ C\left(t\right)=(I_{m}+F\left(t\right))C \end{array}\right.$$

where $\theta_{j}^{u}\left(t\right)$ represents an unknown time-varying parameter associated with a multiplicative actuator attack. The matrices $B_{i}$ and ${\bar{B}}_{ij}$ are constant matrices of compatible dimensions defining the nominal and attack-dependent input dynamics, respectively. Moreover, the matrix $F\left(t\right)\in R^{m\times m}$ is given by:

(68) $$F\left(t\right)=\mathrm{diag}(\theta^{y}\left(t\right))$$

s.t. $\mathrm{diag}(\theta^{y}\left(t\right))$ corresponds to a diagonal matrix with the terms $\theta_{j}^{y}\left(t\right)$ (sensor attacks) on its diagonal. F(t) may be expressed as

(69) $$F\left(t\right)=\sum_{j=1}^{n_{\theta_{y}}}\theta_{j}^{y}\left(t\right)F_{j}$$

Let $n_{\theta_{y}}=m$, and define $F_{j}\in R^{m\times m}$ as diagonal selection matrices with a unit entry at position (j,j) and zeros elsewhere. The index *i* denotes the sensor subject to potential attack. The signals $\theta_{j}^{y}\left(t\right)$ are unknown, time-varying parameters modeling multiplicative sensor attacks.

Actuator deception, including false data injection, is represented by unknown but bounded time-varying parameters $\theta_{j}^{u}\left(t\right)\in \left[\theta_{j}^{u,2},\theta_{j}^{u,1}\right]$, with known bounds. Using the SNT transformation introduced previously, the nonlinear system can be rewritten as:

(70) $$\left\{\begin{array}{ccc} \dot{x}\left(t\right) & = & \sum_{i=1}^{r}\sum_{j=1}^{2^{n_{\theta_{u}}}}\mu_{i}\left(x\left(t\right)\right)\tilde{\mu_{j}}\left(\theta^{u}\left(t\right)\right)\left(A_{i}x\left(t\right)+B_{ij}u\left(t\right)\right)\\ y(t) & = & \sum_{k=1}^{2^{n_{\theta_{y}}}}\bar{\mu_{k}}\left(\theta^{y}\left(t\right)\right){\tilde{C}}_{k}x\left(t\right) \end{array}\right.$$

(71) $$\begin{array}{c} B_{ij}=B_{i}+{\bar{B}}_{ij}\\ {\tilde{C}}_{k}=C+{\bar{F}}_{k}C \end{array}$$

An event-triggered control strategy is considered for the controller design. Accordingly, substituting the control law (64) into the state dynamics (70) leads to:

(72) $$\left\{\begin{array}{ccc} \dot{x}\left(t\right) & = & \sum_{i=1}^{r}\sum_{j=1}^{2^{n_{\theta_{u}}}}\sum_{l=1}^{3^{n_{u}}}\mu_{i}\left(x\left(t\right)\right)\tilde{\mu_{j}}\left(\theta^{u}\left(t\right)\right)\mu_{l}\left(u\left(t\right)\right)\left(A_{i}x\left(t\right)+B_{ij}\Lambda_{l}u\left(t\right)+B_{ij}\left(I_{n_{u}}-\Lambda_{l}\right)u\left(t_{k}\right)\right)\\ y(t) & = & \sum_{k=1}^{2^{n_{\theta_{y}}}}\bar{\mu_{k}}\left(\theta^{y}\left(t\right)\right){\tilde{C}}_{k}x\left(t\right) \end{array}\right.$$

From the global polytopic model (72), a state observer is then designed in the presence of actuator and sensor data deception. An $L_{2}$ attenuation framework is adopted to attenuate the effect of malicious inputs on both state estimation and attack reconstruction errors. The resulting observer dynamics are given by:

(73) $$\left\{\begin{array}{ccc} \dot{\hat{x}}\left(t\right) & = & \sum_{i=1}^{r}\sum_{j=1}^{2^{n_{\theta_{u}}}}\sum_{l=1}^{3^{n_{u}}}\mu_{i}\left(\hat{x}\left(t\right)\right)\tilde{\mu_{j}}\left(\hat{\theta^{u}}\left(t\right)\right)\mu_{l}\left(u\left(t\right)\right)(A_{i}x\left(t\right)+B_{ij}\Lambda_{l}u\left(t\right)\\ \; & \; & +B_{ij}\left(I_{n_{u}}-\Lambda_{l}\right)u\left(t_{k}\right)+L_{ij}\left(y\left(t\right)-\hat{y}\left(t\right)\right))\\ \dot{\hat{\theta^{u}}}\left(t\right) & = & \sum_{i=1}^{r}\sum_{j=1}^{2^{n_{\theta_{u}}}}\mu_{i}\left(\hat{x}\left(t\right)\right)\tilde{\mu_{j}}\left(\hat{\theta^{u}}\left(t\right)\right)\left(K_{ij}^{u}\left(y\left(t\right)-\hat{y}\left(t\right)\right)-\alpha_{ij}^{u}{\hat{\theta }}^{u}\left(t\right)\right)\\ \dot{\hat{\theta^{y}}}\left(t\right) & = & \sum_{i=1}^{r}\sum_{k=1}^{2^{n_{\theta_{y}}}}\mu_{i}\left(\hat{x}\left(t\right)\right)\bar{\mu_{k}}\left(\hat{\theta^{y}}\left(t\right)\right)\left(K_{ik}^{y}\left(y\left(t\right)-\hat{y}\left(t\right)\right)-\alpha_{ik}^{y}\hat{\theta^{y}}\left(t\right)\right)\\ \hat{y}\left(t\right) & = & \sum_{k=1}^{2^{n_{\theta_{y}}}}\bar{\mu_{k}}\left(\hat{\theta^{y}}\left(t\right)\right){\tilde{C}}_{k}\hat{x}\left(t\right) \end{array}\right.$$

where $L_{ij}\in R^{n_{x}\times m}$, $K_{ij}^{u}\in R^{n\times m}$, $\alpha_{ij}^{u}\in R^{n\times n}$, $K_{ik}^{y}\in R^{m\times m}$, and $\alpha_{ik}^{y}\in R^{m\times m}$ are obtained via LMI-based synthesis conditions guaranteeing asymptotic convergence of the estimation errors. Let the state and deception estimation errors $e_{x}\left(t\right)$, $e_{\theta^{u}}\left(t\right)$, and $e_{\theta^{y}}\left(t\right)$ be defined as:

(74) $$\begin{array}{ccc} e_{x}\left(t\right) & = & x\left(t\right)-\hat{x}\left(t\right)\\ e_{\theta^{u}}\left(t\right) & = & \theta^{u}\left(t\right)-\hat{\theta^{u}}\left(t\right)\\ e_{\theta^{y}}\left(t\right) & = & \theta^{y}\left(t\right)-\hat{\theta^{y}}\left(t\right) \end{array}$$

Using the convex-sum property of the weighting functions and following [25], system (70) is equivalently rewritten to enable the derivation of the estimation error dynamics as:

(75) $$\left\{\begin{array}{c} \dot{x}\left(t\right)=\sum_{i=1}^{r}\sum_{j=1}^{2^{n_{\theta_{u}}}}\sum_{l=1}^{3^{n_{u}}}(\mu_{i}\left(\hat{x}\left(t\right)\right)\tilde{\mu_{j}}\left(\hat{\theta^{u}}\left(t\right)\right)\mu_{l}\left(u\left(t\right)\right)\\ (A_{i}x\left(t\right)+B_{ij}\Lambda_{l}u\left(t\right)+B_{ij}\left(I_{n_{u}}-\Lambda_{l}\right)u\left(t_{k}\right))+\\ \alpha_{ij}\left(t\right)\left(A_{i}x\left(t\right)+B_{ij}\Lambda_{l}u\left(t\right)+B_{ij}\left(I_{n_{u}}-\Lambda_{l}\right)u\left(t_{k}\right)\right)))\\ y\left(t\right)=\sum_{k=1}^{2^{n_{\theta_{y}}}}\left[\bar{\mu_{k}}\left(\hat{\theta^{y}}\left(t\right)\right){\tilde{C}}_{k}x\left(t\right)+\bar{\alpha_{k}}\left(t\right){\tilde{C}}_{k}x\left(t\right)\right] \end{array}\right.$$

where $\alpha_{ij}\left(t\right)$ and $\bar{\alpha_{k}}\left(t\right)$ are defined by the following equations:

(76) $$\alpha_{ij}\left(t\right)=\mu_{i}\left(x\left(t\right)\right)\tilde{\mu_{j}}\left(\theta^{u}\left(t\right)\right)-\mu_{i}\left(\hat{x}\left(t\right)\right)\tilde{\mu_{j}}\left(\hat{\theta^{u}}\left(t\right)\right)$$

(77) $$\bar{\alpha_{k}}\left(t\right)=\bar{\mu_{k}}\left(\theta^{y}\left(t\right)\right)-\bar{\mu_{k}}\left(\hat{\theta^{y}}\left(t\right)\right)$$

and satisfy the inequalities:

(78) $$-1\leq \alpha_{ij}\left(t\right)\leq 1,-1\leq \bar{\alpha_{k}}\left(t\right)\leq 1$$

Representation (75) enables a direct derivation of the estimation error dynamics, since the state and output are expressed exclusively in terms of the weighting functions $\mu_{i}\left(\hat{x}\left(t\right)\right)$, ${\tilde{\mu }}_{j}\left({\hat{\theta }}^{u}\left(t\right)\right)$, and ${\bar{\mu }}_{k}\left({\hat{\theta }}^{y}\left(t\right)\right)$.

Let us define now:

(79) $$\Delta A\left(t\right)=\sum_{i=1}^{r}\sum_{j=1}^{2^{n_{\theta_{u}}}}\alpha_{ij}\left(t\right)A_{i}=A\Sigma \left(t\right)E_{A}$$

(80) $$\Delta B\left(t\right)=\sum_{i=1}^{r}\sum_{j=1}^{2^{n_{\theta_{u}}}}\alpha_{ij}\left(t\right)B_{ij}=B\Sigma \left(t\right)E_{B}$$

(81) $$\Delta C\left(t\right)=\sum_{k=1}^{2^{n_{\theta_{y}}}}\bar{\alpha_{k}}\left(t\right){\tilde{C}}_{k}=C\bar{\Sigma }\left(t\right)E_{C}$$

with

(82) $$A=\left[\begin{array}{ccc} \underset{2^{n_{\theta_{u}}}times}{\underset{︸}{\begin{array}{ccc} A_{1} & \ldots & A_{1} \end{array}}} & \ldots & \underset{2^{n_{\theta_{u}}}times}{\underset{︸}{\begin{array}{ccc} A_{r} & \ldots & A_{r} \end{array}}} \end{array}\right]$$

(83) $$B=\left[\begin{array}{ccc} B_{11} & \ldots & B_{r2^{n}} \end{array}\right]$$

(84) $$C=\left[\begin{array}{ccc} {\tilde{C}}_{1} & \ldots & {\tilde{C}}_{2^{n_{\theta_{y}}}} \end{array}\right]$$

(85) $$\begin{array}{c} \Sigma \left(t\right)=\mathrm{diag}(\alpha_{11}\left(t\right),\ldots ,\alpha_{r2^{n}}\left(t\right)),\\ \bar{\Sigma }\left(t\right)=\mathrm{diag}\left(\bar{\alpha_{1}}\left(t\right),\ldots ,\bar{\alpha_{2^{n_{\theta_{y}}}}}\left(t\right)\right) \end{array}$$

(86) $$\begin{array}{c} E_{A}={\left[\begin{array}{ccc} I_{n_{x}} & \ldots & I_{n_{x}} \end{array}\right]}^{T},\;E_{B}={\left[\begin{array}{ccc} I_{n_{u}} & \ldots & I_{n_{u}} \end{array}\right]}^{T}\\ E_{C}={\left[\begin{array}{ccc} I_{2^{m}} & \ldots & I_{2^{m}} \end{array}\right]}^{T} \end{array}$$

Thanks to (76), (77), (85) and the properties in (78):

(87) $$\Sigma^{T}\left(t\right)\Sigma \left(t\right)\leq I,\;{\bar{\Sigma }}^{T}\left(t\right)\bar{\Sigma }\left(t\right)\leq I$$

Using the above definitions (79)–(86), system (75) is then written as an uncertain polytopic fuzzy T-S system given by the system equations:

(88) $$\left\{\begin{array}{ccc} \dot{x}\left(t\right) & = & \sum_{i=1}^{r}\sum_{j=1}^{2^{n_{\theta_{u}}}}\sum_{l=1}^{3^{n_{u}}}\mu_{i}\left(\hat{x}\left(t\right)\right)\tilde{\mu_{j}}\left(\hat{\theta^{u}}\left(t\right)\right)\mu_{l}\left(u\left(t\right)\right)(\left(A_{i}+\Delta A\left(t\right)\right)x\left(t\right)+\left(B_{ij}+\Delta B\left(t\right)\right)\\ \; & \; & \left(\Lambda_{l}u\left(t\right)+\left(I_{n_{u}}-\Lambda_{l}\right)u\left(t_{k}\right)\right)\\ y(t) & = & \sum_{k=1}^{2^{n_{\theta_{y}}}}\bar{\mu_{k}}\left(\hat{\theta^{y}}\left(t\right)\right)\left({\tilde{C}}_{k}+\Delta C\left(t\right)\right)x\left(t\right) \end{array}\right.$$

From Equations (74) and (88), the estimation errors dynamic is then given by:

(89) $$\left\{\begin{array}{ccc} {\dot{e}}_{x}\left(t\right) & = & \sum_{i=1}^{r}\sum_{j=1}^{2^{n_{\theta_{u}}}}\sum_{l=1}^{3^{n_{u}}}\sum_{k=1}^{2^{n_{\theta_{y}}}}\mu_{i}\left(\hat{x}\left(t\right)\right)\tilde{\mu_{j}}\left(\hat{\theta^{u}}\left(t\right)\right)\mu_{l}\left(u\left(t\right)\right)\bar{\mu_{k}}\left(\hat{\theta^{y}}\left(t\right)\right)(\left(A_{i}-L_{ij}{\tilde{C}}_{k}\right)e_{x}\left(t\right)+(\Delta A\left(t\right)\\ & \; & -L_{ij}\Delta C\left(t\right))x\left(t\right)-\Delta B\left(t\right)\Lambda_{l}\delta \left(t\right)+\Delta B\left(t\right)u\left(t_{k}\right))\\ {\dot{e}}_{\theta^{u}}\left(t\right) & = & \sum_{i=1}^{r}\sum_{j=1}^{2^{n_{\theta_{u}}}}\sum_{k=1}^{2^{n_{\theta_{y}}}}\mu_{i}\left(\hat{x}\left(t\right)\right)\tilde{\mu_{j}}\left(\hat{\theta^{u}}\left(t\right)\right)\bar{\mu_{k}}\left(\hat{\theta^{y}}\left(t\right)\right)\\ \; & \; & \left(-K_{ij}^{u}{\tilde{C}}_{k}e_{x}\left(t\right)-\alpha_{ij}^{u}e_{\theta^{u}}\left(t\right)-K_{ij}^{u}\Delta C\left(t\right)x\left(t\right)+\alpha_{ij}^{u}\theta^{u}\left(t\right)+\dot{\theta^{u}}\left(t\right)\right)\\ {\dot{e}}_{\theta^{y}}\left(t\right) & = & \sum_{i=1}^{r}\sum_{k=1}^{2^{n_{\theta_{y}}}}\mu_{i}\left(\hat{x}\left(t\right)\right)\bar{\mu_{k}}\left(\hat{\theta^{y}}\left(t\right)\right)\\ \; & \; & \left(-K_{ik}^{y}{\tilde{C}}_{k}e_{x}\left(t\right)-\alpha_{ik}^{y}e_{\theta^{y}}\left(t\right)-K_{ik}^{y}\Delta C\left(t\right)x\left(t\right)+\alpha_{ik}^{y}\theta^{y}\left(t\right)+\dot{\theta^{y}}\left(t\right)\right) \end{array}\right.$$

Let us now consider the augmented vectors $e_{a}\left(t\right)$ and ω(t), such that:

(90) $$e_{a}\left(t\right)=\left(\begin{array}{c} e_{x}\left(t\right)\\ e_{\theta }^{u}\left(t\right)\\ e_{\theta }^{y}\left(t\right) \end{array}\right),\;\omega \left(t\right)=\left(\begin{array}{c} x(t)\\ \theta^{u}\left(t\right)\\ \theta^{y}\left(t\right)\\ {\dot{\theta }}^{u}\left(t\right)\\ {\dot{\theta }}^{y}\left(t\right)\\ \delta (t)\\ u(t) \end{array}\right)$$

From (89) and (90), it follows:

(91) $$\begin{array}{ccc} {\dot{e}}_{a}\left(t\right) & = & \sum_{i=1}^{r}\sum_{j=1}^{2^{n_{\theta_{u}}}}\sum_{l=1}^{3^{n_{u}}}\sum_{k=1}^{2^{n_{\theta_{y}}}}\mu_{i}\left(\hat{x}\left(t\right)\right)\tilde{\mu_{j}}\left(\hat{\theta^{u}}\left(t\right)\right)\mu_{l}\left(u\left(t\right)\right)\bar{\mu_{k}}\left(\hat{\theta^{y}}\left(t\right)\right)\left(\Phi_{ijk}e_{a}\left(t\right)+\Psi_{ijk}\left(t\right)\omega \left(t\right)\right) \end{array}$$

with

(92) $$\begin{array}{c} \Phi_{ijk}=\left(\begin{array}{ccc} A_{i}-L_{ij}{\tilde{C}}_{k} & 0 & 0\\ -K_{ij}^{u}{\tilde{C}}_{k} & -\alpha_{ij}^{u} & 0\\ -K_{ik}^{y}{\tilde{C}}_{k} & 0 & -\alpha_{ik}^{y} \end{array}\right)\\ \Psi_{ijk}\left(t\right)=\\ \left(\begin{array}{ccccccc} \Delta A(t) & 0 & 0 & 0 & 0 & -\Delta B(t) & 0\\ -K_{ij}^{u}\Delta C\left(t\right) & \alpha_{ij}^{u} & 0 & I & 0 & 0 & 0\\ -K_{ik}^{u}\Delta C\left(t\right) & 0 & \alpha_{ik}^{y} & 0 & I & \Delta B(t) & \Delta B(t) \end{array}\right) \end{array}$$

Considering (91), the objective is to design a simultaneous state and attack observer ensuring a minimal $L_{2}$ gain from ω(t) to $e_{a}\left(t\right)$. The computation of the corresponding gains is detailed in the following theorem.

**Remark** **5.** *To apply the considered criterion, a minimal $L_{2}$ attenuation is imposed between the augmented estimation error vector, $e_{a}\left(t\right)$, and the external input ω(t). The signal ω(t) is assumed to have finite energy. For the stable case considered, and accounting for the non-continuous nature of stealthy attacks, this assumption holds.*

**Theorem** **1.** *A state and actuator/sensor data deception observer (73) for the nonlinear system (66) guarantees an $L_{2}$ gain from ω(t) to $e_{a}\left(t\right)$ bounded by β>0, provided that there exist symmetric positive definite matrices $P_{1}$, $P_{2}$, $P_{3}$, positive matrices $\Gamma_{l}$ (l=1,…,6), matrices ${\bar{\alpha }}_{ij}^{u}$, ${\bar{\alpha }}_{ik}^{y}$, $F_{ij}^{u}$, $F_{ik}^{y}$, $R_{ij}$, and positive scalars β, $\lambda_{A}$, $\lambda_{B}$, $\lambda_{1C}$, $\lambda_{2C}$ satisfying the optimization problem (93) under the LMI constraints (94) and (95)*

(93) $$\min_{\left\{P_{1},P_{2},P_{3},R_{ij},F_{ij}^{u},F_{ik}^{y},{\bar{\alpha }}_{ij}^{u},{\bar{\alpha }}_{ik}^{y},Gamma_{l},\lambda_{A},\lambda_{B},\lambda_{1C},\lambda_{2C}\right\}}\beta$$

*for i=1,…,r, $j=1,2^{n_{\theta_{u}}}$ and $k=1,2^{n_{\theta_{y}}}$*

(94) $$\Gamma_{l}<\beta I\;\mathrm{for}\;l=1,\ldots ,6$$

(95) $$\left[\begin{array}{c} \left(\begin{array}{ccccccccccccccc} Q_{ijk}^{11}\; & -{\tilde{C}}_{k}^{T}{F_{ij}^{u}}^{T}\; & -{\tilde{C}}_{k}^{T}{F_{ik}^{y}}^{T}\; & 0\; & 0\; & 0\; & 0\; & 0\; & 0\; & \;0 & P_{1}A\; & P_{1}B\; & \;0 & 0\; & \;0\\ {}*\; & Q_{ij}^{22}\; & 0\; & 0\; & {\bar{\alpha }}_{ij}^{u}\; & 0\; & P_{2}\; & 0\; & 0\; & \;0 & 0\; & 0\; & \;0 & F_{ij}^{u}C & \;0\\ {}*\; & *\; & Q_{ik}^{33}\; & 0\; & 0\; & {\bar{\alpha }}_{ik}^{y}\; & 0\; & P_{3}\; & \;0 & 0\; & 0\; & 0\; & \;P_{1}B & 0\; & \;F_{ik}^{y}C\\ {}*\; & *\; & *\; & Q^{44}\; & 0\; & 0\; & 0\; & 0\; & 0\; & \;0 & 0\; & 0\; & \;0 & 0\; & \;0\\ {}*\; & *\; & *\; & *\; & -\Gamma_{2}\; & 0\; & 0\; & 0\; & 0\; & \;0 & 0\; & 0\; & \;0 & 0\; & \;0\\ {}*\; & *\; & *\; & *\; & *\; & -\Gamma_{3}\; & 0\; & 0\; & 0\; & \;0 & 0\; & 0\; & \;0 & 0\; & \;0\\ {}*\; & *\; & *\; & *\; & *\; & *\; & -\Gamma_{4}\; & 0\; & 0\; & \;0 & 0\; & 0\; & \;0 & 0\; & \;0\\ {}*\; & *\; & *\; & *\; & *\; & *\; & *\; & -\Gamma_{5}\; & 0\; & \;0 & 0\; & 0\; & \;0 & 0\; & \;0\\ {}*\; & *\; & *\; & *\; & *\; & *\; & *\; & *\; & -\Gamma_{6}+\lambda_{B}E_{B}^{T}E_{B}\; & \;0 & 0\; & 0\; & \;0 & 0\; & \;0\\ {}*\; & *\; & *\; & *\; & *\; & *\; & *\; & *\; & *\; & -\Gamma_{7}+\lambda_{B}E_{B}^{T}E_{B}\; & 0\; & 0\; & \;0 & 0\; & \;0\\ {}*\; & *\; & *\; & *\; & *\; & *\; & *\; & *\; & *\; & \;* & -\lambda_{A}I\; & 0\; & \;0 & 0\; & \;0\\ {}*\; & *\; & *\; & *\; & *\; & *\; & *\; & *\; & *\; & \;* & *\; & -\lambda_{B}I\; & \;0 & 0\; & \;0\\ {}*\; & *\; & *\; & *\; & *\; & *\; & *\; & *\; & *\; & \;* & *\; & *\; & \;-\lambda_{B}I & 0\; & \;0\\ {}*\; & *\; & *\; & *\; & *\; & *\; & *\; & *\; & *\; & \;* & *\; & *\; & \;0 & -\lambda_{1C}I\; & \;0\\ {}*\; & *\; & *\; & *\; & *\; & *\; & *\; & *\; & *\; & \;* & *\; & *\; & \;0 & 0\; & \;-\lambda_{2C}I \end{array}\right)<0 \end{array}\right]$$

*with*

(96) $$\begin{array}{c} Q_{ijk}^{11}=P_{1}A_{i}+A_{i}^{T}P_{1}-R_{i}{\tilde{C}}_{j}-{\tilde{C}}_{j}^{T}R_{i}^{T}+I_{n_{x}}\\ Q_{ij}^{22}=-{\bar{\alpha }}_{ij}^{u}-{{\bar{\alpha }}_{ij}^{u}}^{T}+I\\ Q_{ik}^{33}=-{\bar{\alpha }}_{ik}^{y}-{{\bar{\alpha }}_{ik}^{y}}^{T}+I\\ Q^{44}=-\Gamma_{1}+\lambda_{A}E_{A}^{T}E_{A} \end{array}$$

*The observer gains are given by*

(97) $$\left\{\begin{array}{c} L_{ij}=P_{1}^{-1}R_{ij}\\ K_{ij}^{u}=P_{2}^{-1}F_{ij}^{u}\\ K_{ik}^{y}=P_{3}^{-1}F_{ik}^{y}\\ \alpha_{ij}^{u}=P_{2}^{-1}{\bar{\alpha }}_{ij}^{u}\\ \alpha_{ik}^{y}=P_{3}^{-1}{\bar{\alpha }}_{ik}^{y} \end{array}\right.$$

**Proof.**  Let us consider the following quadratic Lyapunov function:

(98) $$V\left(e_{a}\left(t\right)\right)=e_{a}^{T}\left(t\right)Pe_{a}\left(t\right),\;P=P^{T}>0$$

Using (91), its time derivative is given by

(99) $$\begin{array}{c} \dot{V}\left(e_{a}\left(t\right)\right)=\sum_{i=1}^{r}\sum_{j=1}^{2^{n_{\theta_{u}}}}\sum_{k=1}^{2^{n_{\theta_{y}}}}\mu_{i}\left(\hat{x}\left(t\right)\right)\tilde{\mu_{j}}\left(\hat{\theta^{u}}\left(t\right)\right)\bar{\mu_{k}}\left(\hat{\theta^{y}}\left(t\right)\right)\\ \left[e_{a}^{T}\left(t\right)\left({\left(\Phi_{ij}\right)}^{T}P+P\Phi_{ij}\right)e_{a}\left(t\right)+e_{a}^{T}\left(t\right)P\Psi_{i}\left(t\right)\omega \left(t\right)\right.\\ \left.+\omega^{T}\left(t\right)\Psi_{i}^{T}\left(t\right)Pe_{a}\left(t\right)\right] \end{array}$$

It is known that $e_{a}\left(t\right)$ asymptotically converges to zero for ω(t)=0, and that the $L_{2}$ gain from ω(t) to $e_{a}\left(t\right)$ is upper-bounded by β provided that the following inequality holds:

(100) $$\dot{V}\left(e_{a}\left(t\right)\right)+e_{a}^{T}\left(t\right)e_{a}\left(t\right)-\omega^{T}\left(t\right)\Gamma \omega \left(t\right)<0$$

with

(101) $$\Gamma =\mathrm{diag}\left(\Gamma_{l}\right),\;\Gamma_{l}<\beta \;I,\;\mathrm{for}\;l=1,\ldots ,7$$

An appropriate choice of Γ enables to attenuate the transfer from some components of ω(t) to $e_{a}\left(t\right)$.

From (99), (100) becomes:

(102) $$\begin{array}{c} \sum_{i=1}^{r}\sum_{j=1}^{2^{n_{\theta_{u}}}}\sum_{k=1}^{2^{n_{\theta_{y}}}}\mu_{i}\left(\hat{x}\left(t\right)\right)\tilde{\mu_{j}}\left(\hat{\theta^{u}}\left(t\right)\right)\bar{\mu_{k}}\left(\hat{\theta^{y}}\left(t\right)\right){\left(\begin{array}{c} e_{a}\left(t\right)\\ \omega (t) \end{array}\right)}^{T}\\ \left(\begin{array}{cc} \Phi_{ij}^{T}P+P\Phi_{ij}+I & P\Psi_{i}\left(t\right)\\ \Psi_{i}^{T}\left(t\right)P & -\Gamma \end{array}\right)\left(\begin{array}{c} e_{a}\left(t\right)\\ \omega (t) \end{array}\right)<0 \end{array}$$

For a chosen structure of the Lyapunov matrix *P* (diagonal),

(103) $$P=\mathrm{diag}(P_{1},P_{2},P_{3})$$

With the change of variables defined in (97), and using the decompositions (79)–(81), together with the properties in (87), Schur’s complement, and the following lemma:

**Lemma** **4** ([28])**.** *Consider two matrices X and Y with appropriate dimensions, a time-varying matrices Δ(t) and a positive scalar ε. The following property is verified*

(104) $$X^{T}\Delta^{T}\left(t\right)Y+Y^{T}\Delta \left(t\right)X\leq \varepsilon X^{T}X+\varepsilon^{-1}Y^{T}Y$$

*for $\Delta^{T}\left(t\right)\Delta \left(t\right)\leq I$.*

Following the approach in [22,25], Lyapunov stability with an $L_{2}$ gain from ω(t) to $e_{a}\left(t\right)$ is established by solving the optimization problem (93) under the LMI constraints (94) and (95).

Indeed, the negativity of condition (102), resulting from the convex-sum property of the weighting functions and the quadratic structure of the vector $\left(e_{a}\left(t\right),\;\omega \left(t\right)\right)$, is ensured if, by Lyapunov arguments,

(105) $$\left(\begin{array}{cc} \Phi_{ij}^{T}P+P\Phi_{ij}+I & P\Psi_{i}\left(t\right)\\ \Psi_{i}^{T}\left(t\right)P & -\Gamma \end{array}\right)<0.$$

Substituting $\Phi_{ijk}\left(t\right)$ and $\Psi_{ijk}$ with their expressions in (92), and applying Lemma 1, Schur’s complement, and the congruence transformation principle, the result follows, which completes the proof. □

**Figure 1:**
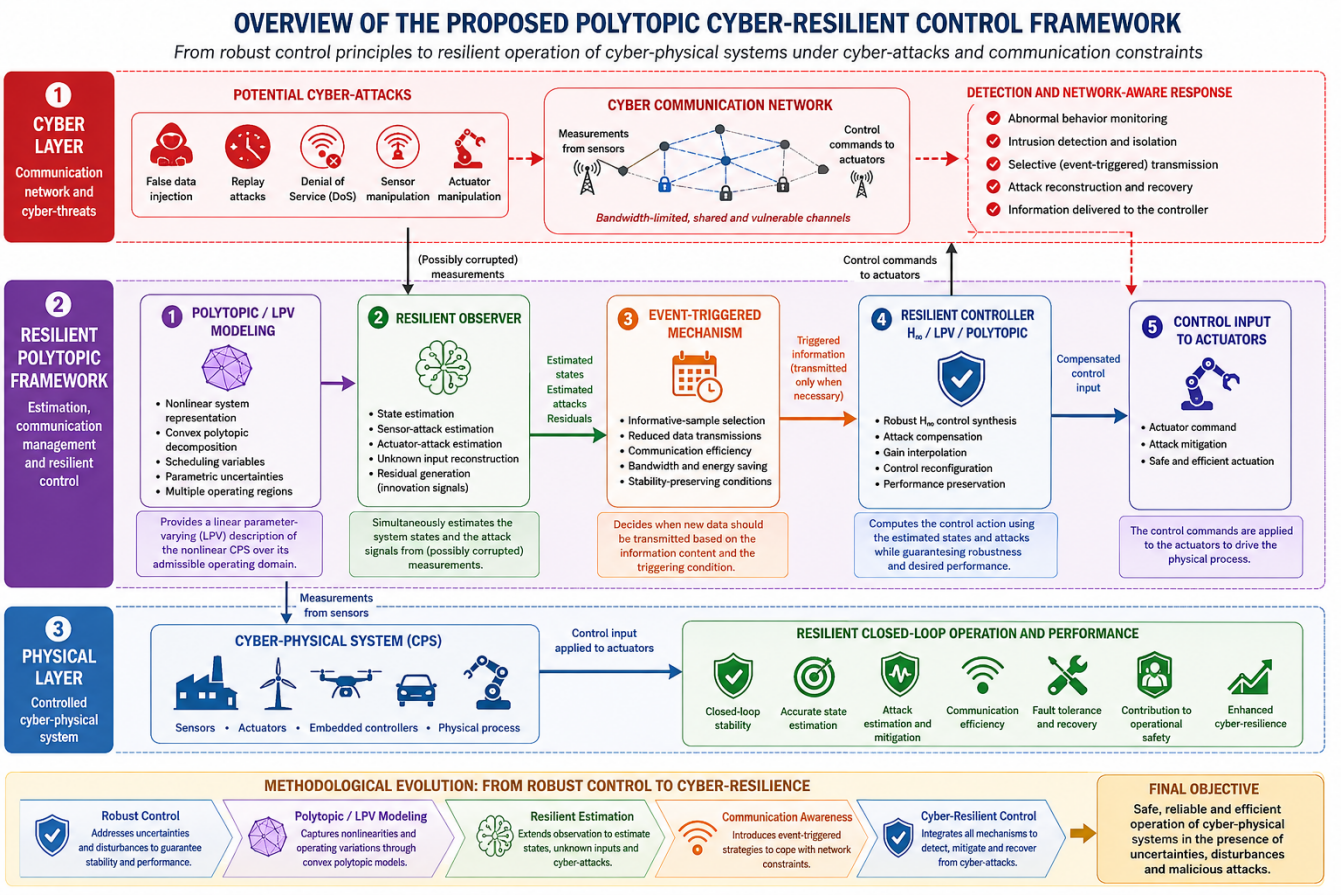
Overview architecture of the proposed polytopic cyber-resilient framework.

The following Figure 1 illustrates the general architecture and methodological evolution of the proposed cyber-resilient polytopic framework. The nonlinear cyber-physical system is first represented through an LPV or polytopic model that captures its operating conditions, nonlinearities and uncertainties. A resilient observer uses the available measurements to simultaneously estimate the system states and reconstruct malicious sensor and actuator attacks. The resulting estimates are processed through an event-triggering mechanism so that only sufficiently informative data are transmitted over the communication network. The resilient controller subsequently combines robust $H_{\infty }$ performance with attack-compensation and gain-reconfiguration mechanisms to generate an appropriate control action. This closed-loop architecture contributes to operational safety by preserving stability, bounded estimation errors and acceptable performance despite malicious attacks and communication constraints. The lower part of the figure emphasizes that the transition from robust control to cyber-resilience represents a progressive methodological enrichment rather than an opposition between the two concepts. Thus, attack detection is not treated as an isolated alarm-generation task; it is directly integrated into the estimation and control loop so that the system can respond to the detected intrusion.

## 6. Illustrative Example and Results

The proposed approach is illustrated using a simplified biological wastewater treatment plant model. The dynamics are described by two state variables $x_{1}\left(t\right)$ and $x_{2}\left(t\right)$, representing biomass and substrate concentrations, respectively. The control input u(t) corresponds to the residence (dwell) time in the reactor, while the measured output is the biomass concentration, given by $y\left(t\right)=x_{1}\left(t\right)$.

It is important to highlight that the simulation example considered in this work is intended as an illustrative validation of the proposed theoretical framework. The choice of a biological wastewater treatment process was motivated by its well-established nonlinear dynamics and its suitability for evaluating the effectiveness of the proposed observer and resilient control design. Since the proposed synthesis methodology is independent of the specific application, the conclusions drawn from this example are not limited to this benchmark. Nevertheless, the validation of the proposed framework on larger-scale networked cyber-physical systems explicitly accounting for communication constraints, such as network-induced delays, packet losses, and coordinated cyber-attacks, as well as the assessment of additional communication performance indicators, remains an important perspective for future investigations.

### 6.1. LPV Formulation of the Process Dynamics

As a first step, the nonlinear system (106) is rewritten in a polytopic form. Following [24] and under appropriate assumptions, several simplifications can be introduced, yielding the nonlinear model:

(106) $$\left\{\begin{array}{c} {\dot{x}}_{1}\left(t\right)=\frac{ax_{1}\left(t\right)x_{2}\left(t\right)}{x_{2}\left(t\right)+b}-x_{1}\left(t\right)u\left(t\right)\\ {\dot{x}}_{2}\left(t\right)=-\frac{cax_{1}\left(t\right)x_{2}\left(t\right)}{x_{2}\left(t\right)+b}+\left(d-x_{2}\left(t\right)\right)u\left(t\right) \end{array}\right.$$

where *a*, *b*, *c*, and *d* are known parameters.

To capture the nonlinearities, a Sector Nonlinearity approach is adopted with premise variables defined as:

(107) $$\rho_{1}\left(t\right)=-u\left(t\right),\;\rho_{2}\left(t\right)=\frac{ax_{1}\left(t\right)}{x_{2}\left(t\right)+b}.$$

Based on (106) and (107), the following quasi-LPV representation is obtained:

(108) $$\dot{x}\left(t\right)=\left(\begin{array}{cc} \rho_{1}\left(t\right) & \rho_{2}\left(t\right)\\ 0 & -c\rho_{2}\left(t\right)+\rho_{1}\left(t\right) \end{array}\right)x\left(t\right)+\left(\begin{array}{c} 0\\ d \end{array}\right)u\left(t\right).$$

Since the LPV representation is defined over a compact region of the state space, the extrema of $\rho_{1}\left(t\right)$ and $\rho_{2}\left(t\right)$ can be determined from the admissible variation range of u(t), leading to $\rho_{1}\left(t\right)\in \left[-1,-0.2\right]$ and $\rho_{2}\left(t\right)\in \left[0.004,15\right]$.

Applying the convex polytopic transformation, two partitions for each premise variable are defined:

(109) $$\left\{\begin{array}{c} \rho_{1}\left(t\right)=\varrho_{11}\left(\rho_{1}\right)\rho_{1}^{2}+\varrho_{12}\left(\rho_{1}\right)\rho_{1}^{1}\\ \rho_{2}\left(t\right)=\varrho_{21}\left(\rho_{2}\right)\rho_{2}^{2}+\varrho_{22}\left(\rho_{2}\right)\rho_{2}^{1} \end{array}\right.$$

(110) $$\begin{array}{c} \mathrm{with}\;\varrho_{11}\left(\rho_{1}\right)=\frac{\rho_{1}\left(t\right)-\rho_{1}^{2}}{\rho_{1}^{1}-\rho_{1}^{2}},\;\varrho_{12}\left(\rho_{1}\right)=\frac{\rho_{1}^{1}-\rho_{1}\left(t\right)}{\rho_{1}^{1}-\rho_{1}^{2}}\\ \varrho_{21}\left(\rho_{2}\right)=\frac{\rho_{2}\left(t\right)-\rho_{2}^{2}}{\rho_{2}^{1}-\rho_{2}^{2}},\;\varrho_{22}\left(\rho_{2}\right)=\frac{\rho_{2}^{1}-\rho_{2}\left(t\right)}{\rho_{2}^{1}-\rho_{2}^{2}} \end{array}$$

where the scalars $\rho_{1}^{1}$, $\rho_{1}^{2}$, $\rho_{2}^{1}$ and $\rho_{2}^{2}$ are defined as

(111) $$\begin{array}{c} \rho_{1}^{1}=\max_{u}\rho_{1}\left(t\right),\;\rho_{1}^{2}=\min_{u}\rho_{1}\left(t\right)\\ \rho_{2}^{1}=\max_{x}\rho_{2}\left(t\right),\;\rho_{2}^{2}=\min_{x}\rho_{2}\left(t\right) \end{array}$$

The resulting submodels are characterized by the triplets $\left(A_{i},B_{i},C\right)$, i=1,2,3,4. Based on the definitions of $\rho_{1}$ and $\rho_{2}$, all input matrices are identical and given by

$$B={\left[\begin{array}{cc} 0 & d \end{array}\right]}^{T}.$$

The output matrix is defined as $C=\left[\begin{array}{cc} 1 & 0 \end{array}\right]$, while the system matrices $A_{i}$ are given by:

$$\begin{array}{c} A_{1}=\left(\begin{array}{cc} \rho_{1}^{1} & \rho_{2}^{1}\\ 0 & -c\rho_{2}^{1}+\rho_{1}^{1} \end{array}\right),\;A_{2}=\left(\begin{array}{cc} \rho_{1}^{1} & \rho_{2}^{2}\\ 0 & -c\rho_{2}^{2}+\rho_{1}^{1} \end{array}\right)\\ A_{3}=\left(\begin{array}{cc} \rho_{1}^{2} & \rho_{2}^{1}\\ 0 & -c\rho_{2}^{1}+\rho_{1}^{2} \end{array}\right),\;A_{4}=\left(\begin{array}{cc} \rho_{1}^{2} & \rho_{2}^{2}\\ 0 & -c\rho_{2}^{2}+\rho_{1}^{2} \end{array}\right) \end{array}$$

The weighting functions $\mu_{i}\left(t\right)$ are defined by the following equations:

(112) $$\begin{array}{c} \mu_{1}\left(t\right)\;=\varrho_{11}\left(\rho_{1}\left(t\right)\right)\varrho_{21}\left(\rho_{2}\left(t\right)\right),\mu_{2}\left(t\right)\;=\varrho_{11}\left(\rho_{1}\left(t\right)\right)\varrho_{22}\left(\rho_{2}\left(t\right)\right)\\ \mu_{3}\left(t\right)\;=\varrho_{12}\left(\rho_{1}\left(t\right)\right)\varrho_{21}\left(\rho_{2}\left(t\right)\right),\mu_{4}\left(t\right)\;=\varrho_{12}\left(\rho_{1}\left(t\right)\right)\varrho_{22}\left(\rho_{2}\left(t\right)\right) \end{array}$$

Since the polytopic representation is defined over a compact region of the state space, the extrema of $\rho_{1}\left(t\right)$ and $\rho_{2}\left(t\right)$ can be determined from the admissible variation range of u(t), yielding $\rho_{1}\left(t\right)\in \left[-1,-0.2\right]$ and $\rho_{2}\left(t\right)\in \left[0.004,15\right]$.

### 6.2. Actuator/Sensor Data Deception Attack Modeling

Two types of data deception attacks are considered, namely actuator and sensor attacks. These are modeled as bounded multiplicative time-varying faults affecting both actuators and sensors.

In the considered example, the parameter *d* is assumed to be vulnerable to malicious modification. The corresponding actuator attack is represented by a time-varying signal d(t) such that:

(113) d(t)=d+Δd(t)

It can also be written as:

(114) $$d\left(t\right)=d+\theta^{u}\left(t\right)\bar{d},\;\theta^{u}\left(t\right)\in \left[{\theta^{u}}^{2},{\theta^{u}}^{1}\right]$$

The parameters are set as d=2.5, $\bar{d}=2.1$, and ${\theta^{u}}^{2}=-0.1958$, ${\theta^{u}}^{1}=0.1979$. The system parameters are identified as a=0.5, b=0.07, and c=0.7.

Considering actuator attacks, the input matrix *B* admits a polytopic representation involving two submodels, given by:

(115) $$\begin{array}{c} B_{1}=B+{\theta^{u}}^{1}\bar{B},\;B_{2}=B+{\theta^{u}}^{2}\bar{B} \end{array}$$

where $\bar{B}:={\left[\begin{array}{cc} 0 & \bar{d} \end{array}\right]}^{T}$. The weighting functions $\tilde{\mu_{j}}\left(\theta^{u}\left(t\right)\right)$ are defined as given in (7) and (12).

Now, for the sensor attack, it is assumed that a bounded multiplicative sensor fault $\theta^{y}\left(t\right)$ affects the output y(t) such that:

(116) $$y\left(t\right)=\left(1+\theta^{y}\left(t\right)\right)x_{1}\left(t\right)$$

As previously explained, $\theta^{y}\left(t\right)$ can also be written as:

(117) $$\theta^{y}\left(t\right)={{\bar{\mu }}_{1}}^{1}\left(\theta^{y}\left(t\right)\right){\theta^{y}}^{1}+{\bar{\mu }}_{1}^{2}\left(\theta^{y}\left(t\right)\right){\theta^{y}}^{2},\;\theta^{y}\left(t\right)\in \left[{\theta^{y}}^{2},{\theta^{y}}^{1}\right]$$

with ${\theta^{y}}^{2}=0.125$ and ${\theta^{y}}^{1}=0.625$, and where ${{\bar{\mu }}_{1}}^{1}\left(\theta^{y}\left(t\right)\right)$ and ${\bar{\mu }}_{1}^{2}\left(\theta^{y}\left(t\right)\right)$ are defined by (9) and (13).

The polytopic form of the output is then given by:

(118) $$y\left(t\right)=\sum_{k=1}^{2}{\bar{\mu }}_{k}\left(\theta^{y}\left(t\right)\right){\tilde{C}}_{k}x\left(t\right)$$

with ${\tilde{C}}_{1}=\left(\begin{array}{cc} 1+{\theta^{y}}^{2} & 0 \end{array}\right),\;{\tilde{C}}_{2}=\left(\begin{array}{cc} 1+{\theta^{y}}^{1} & 0 \end{array}\right)$.

### 6.3. Simulation Results

For the considered example with simultaneous actuator and sensor attacks, the proposed methodology, combining a robust $H_{\infty }$ framework and event-triggered control, is used to design a simultaneous state and attack observer. The system initial condition is set to $x\left(0\right)=\left(\begin{array}{cc} 0.1 & 1.5 \end{array}\right)$, while the observer is initialized as $\hat{x}\left(0\right)=\left(\begin{array}{cc} 0.09 & 2.3 \end{array}\right)$. The attack estimates are initialized at zero, i.e., ${\hat{\theta }}^{u}\left(0\right)=0$ and ${\hat{\theta }}^{y}\left(0\right)=0$.

**Figure 2:**
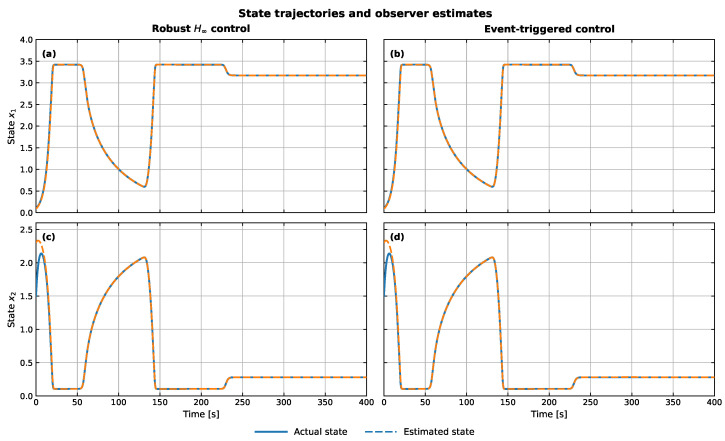
Comparison of the system states and their observer estimates obtained with the robust $H_{\infty }$ and event-triggered control strategies: (**a**,**b**) first state $x_{1}\left(t\right)$ and (**c**,**d**) second state $x_{2}\left(t\right)$. Solid lines represent the actual states, whereas dashed lines denote their estimates.

**Figure 3:**
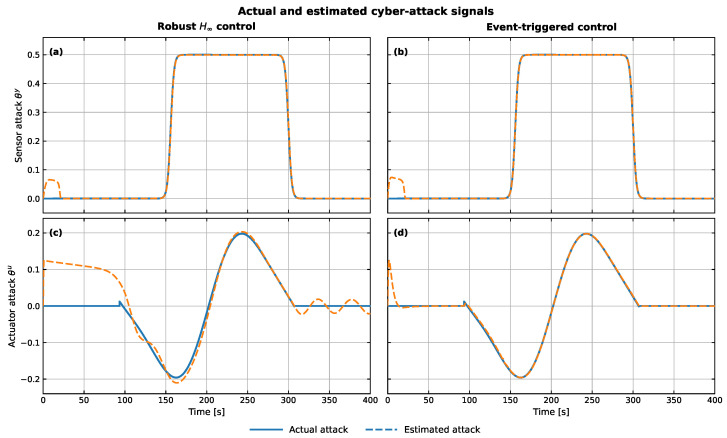
Comparison of the actual and estimated cyber-attack signals obtained with the robust $H_{\infty }$ and event-triggered control strategies: (**a**,**b**) sensor attack $\theta^{y}\left(t\right)$ and (**c**,**d**) actuator attack $\theta^{u}\left(t\right)$. Solid lines represent the actual attacks, whereas dashed lines denote their estimates.

For both strategies, i.e., a robust $H_{\infty }$ output-feedback controller and the event-triggered controller, the state trajectories and their corresponding estimates are presented in Figure 2, whereas the actual and estimated multiplicative actuator and sensor deception attacks are shown in Figure 3.

The figures show clearly that the proposed observer accurately reconstructs both the system states and the time-varying attack signals despite the presence of simultaneous actuator and sensor attacks. Furthermore, the $H_{\infty }$ controller successfully attenuates the impact of the attacks on the closed-loop system, leading to stable system behavior and satisfactory disturbance-rejection performance. The error between the actual and estimated trajectories confirms the effectiveness of the proposed resilient estimation and control scheme.

For the second strategy, which combines the attack observer with an event-triggered controller, it can be observed that the proposed observer preserves an accurate reconstruction of both the system states and the multiplicative actuator/sensor attacks. Despite the reduced number of control updates induced by the event-triggering mechanism, the closed-loop system remains stable and the estimation performance is maintained. These results demonstrate that the proposed event-triggered strategy achieves a satisfactory trade-off between control performance and communication efficiency while preserving resilience against cyber-attacks. The close overlap of the state and time-varying signals trajectories demonstrates that the event-triggered implementation preserves the stabilization, attack attenuation, and estimation capabilities of the nominal $H_{\infty }$ controller while operating under an event-based update mechanism.

From the presented figures, one can see that both approaches accurately reconstruct the system states and malicious attack signals while ensuring closed-loop stability. In particular, the state trajectories obtained with the event-triggered controller match those generated by the robust $H_{\infty }$ controller. For the data deception signals, they indeed closely match, with only negligible deviations observed during the transient phase and final one due to the sample-and-hold implementation inherent to event-triggered control. This simulation result demonstrates that the proposed triggering mechanism successfully preserves the stabilization, attack attenuation, and estimation capabilities of the nominal $H_{\infty }$ controller despite operating with an event-based update scheme. These results indicate that the triggering condition effectively determines when control updates are required, thereby avoiding unnecessary transmissions without compromising resilience or control performance. Consequently, while the robust $H_{\infty }$ controller primarily aims at guaranteeing stability and disturbance/attack attenuation through continuous control updates, the proposed event-triggered strategy preserves these robustness properties while offering a more efficient utilization of communication and computational resources. Overall, the comparison highlights the ability of the proposed resilient event-triggered architecture to reproduce the performance of the nominal $H_{\infty }$ controller, thereby achieving an effective trade-off between closed-loop performance, attack resilience, and resource utilization.

Since the present article is intended as a comprehensive overview of previously published methodologies rather than an original research contribution, the computational performance of each approach has already been investigated and reported in the corresponding original publications, where detailed numerical simulations, computational analyses, and, when available, experimental validations are presented. Consequently, the objective of this overview is not to reproduce these results but to provide a unified methodological perspective on the evolution of the proposed polytopic framework. Moreover, because the reviewed methodologies address different classes of systems, control architectures, and application domains, a unified experimental comparison of computational efficiency would not constitute a fair or scientifically meaningful assessment. Readers interested in implementation aspects and computational performance are referred to the cited original publications for further details.

## 7. Discussion: Toward Advanced CPS Resilience

The proposed framework should not be interpreted merely as a collection of observer and controller synthesis techniques. Its main contribution lies in providing a unified convex modeling paradigm in which nonlinearities, uncertainties, cyber-attacks and communication constraints are represented within a common mathematical structure. This unified representation allows resilience-oriented estimation and control problems to be addressed using a consistent LMI-based design methodology. This representation subsequently supports the joint synthesis of state-and-attack observers, robust $H_{\infty }$ controllers, and event-triggered control mechanisms through a common Lyapunov-based framework. The resulting architecture therefore links four normally separated functions: system representation, attack reconstruction, closed-loop control, and communication-aware control. The manuscript currently describes these complementary strategies, including attack attenuation and simultaneous state-and-attack estimation, together with an event-triggered co-design intended to reduce communication and computational requirements.

In the context of advanced CPS resilience control, the principal advantage of the framework is its ability to provide formal stability and performance guarantees while retaining a structured representation of nonlinear and attack-dependent dynamics. Unlike approaches restricted to residual-based detection, the proposed observer estimates both the physical states and the malicious attack variables, allowing the reconstructed information to be directly exploited by the resilient controller. The event-triggered extension additionally provides an implementation in which information is transmitted only when the triggering condition is satisfied. The approach is therefore particularly relevant when resilience, nonlinear behavior, and communication limitations must be addressed within the same design framework.

Nevertheless, the methodology has several limitations that define its current domain of validity:

- The approach is fully model-based and consequently depends on the availability of a sufficiently representative dynamic model. It does not yet include adaptive or learning capabilities; online parameter adaptation, data-driven estimation, or learning-assisted resilience mechanisms constitute an important direction for future research.
- The attack parameters are assumed to remain within prescribed bounded intervals. The established guarantees therefore apply only inside the considered admissible domain and cannot be extended directly to arbitrary out-of-bound attacks or to adversarial signals specifically designed to exploit unmodeled zero dynamics.
- The number of polytopic vertices may grow rapidly with the number of scheduling variables, attacked channels, and local models. Although this complexity mainly affects the offline synthesis stage, the numerical burden may become restrictive for very large-scale CPSs.
- The LMI/BMI conditions constitute sufficient rather than necessary conditions; therefore, infeasibility does not necessarily imply that no stabilizing resilient observer–controller exists.
- Although multiplicative attacks provide a meaningful representation of gain manipulation, calibration corruption, and partial loss of actuator or sensor effectiveness, extending the framework to additive and hybrid attack models would broaden its applicability.

These limitations naturally open several promising directions for further methodological developments. From a computational perspective, scalability remains a key challenge when addressing increasingly complex cyber-physical systems. Although the online implementation only requires the interpolation of precomputed gains, the offline synthesis complexity increases with the number of scheduling variables, operating regions, and attack channels. Future developments may therefore benefit from advanced vertex-reduction techniques, distributed optimization methods, subsystem decomposition strategies, hierarchical observer structures, and decentralized or multi-agent resilient control architectures. Such approaches would improve the applicability of the framework to large-scale networked CPSs while preserving the advantages of the convex polytopic formulation.

Another important direction concerns the integration of complementary resilience mechanisms. The proposed methodology is fundamentally model-based and relies on the physical dynamics of the process to achieve simultaneous state and attack estimation. However, future resilient CPS architectures are expected to combine model-based estimation with data-driven intelligence. In this context, the proposed observer–controller framework could be coupled with active-defense mechanisms, such as learning-based anomaly detection algorithms capable of identifying previously unseen attack patterns. Rather than replacing model-based observers, these techniques should be viewed as complementary layers within a defense-in-depth strategy, where physics-based estimation provides formal stability and robustness guarantees while data-driven components enhance adaptability and detection capabilities against sophisticated or stealthy cyber-attacks.

Finally, further research should investigate more adaptive resilient control architectures. The current framework assumes known system models and bounded uncertainty domains. Incorporating adaptive estimation techniques, online identification methods, or learning-assisted parameter updates would allow the controller and observer to evolve with changing operating conditions and unknown attack characteristics. Such hybrid model-based and learning-enhanced frameworks represent a promising step toward the next generation of resilient autonomous cyber-physical systems.

## 8. Conclusions and Perspectives

This overview has presented the progressive evolution of the polytopic framework from its foundations in robust control to its recent developments in resilient estimation and control for safety-critical cyber-physical systems. It provides a unified methodological perspective by bringing together the principal methodological developments in robust control, observer design, attack estimation, event-triggered communication, and cyber-resilient control within a common theoretical framework. The overview also highlights the main characteristics of the framework, discusses its current challenges, and identifies several promising research directions, including learning-enhanced resilient control, distributed architectures, and next-generation autonomous cyber-physical systems.

Overall, this overview demonstrates how the polytopic framework has progressively evolved into a unified methodology capable of addressing increasingly complex challenges encountered in modern safety-critical cyber-physical systems while preserving the rigorous model-based foundations required for analysis, estimation, and control.

This overview has examined the application of the polytopic framework to the resilient estimation and control of safety-critical cyber-physical systems subject to data deception attacks. In particular, it has presented two complementary control strategies illustrating the evolution of the proposed methodology. The first is based on a robust $H_{\infty }$ control framework that guarantees closed-loop stability while attenuating the effects of disturbances and cyber-attacks. The second extends this framework through an event-triggered resilient control strategy that preserves the robustness properties of the nominal controller while reducing the frequency of control updates. In both approaches, dedicated observers are designed to simultaneously estimate the system states and the malicious attack signals.

The reviewed results demonstrate the capability of the proposed framework to accurately reconstruct both the system states and the attack signals in the presence of time-varying multiplicative actuator and sensor attacks. They also show that the robust and event-triggered control strategies preserve closed-loop stability while ensuring satisfactory disturbance attenuation and resilience against cyber-attacks. Furthermore, the event-triggered implementation achieves a dynamic behavior comparable to that of the robust $H_{\infty }$ controller while significantly reducing communication and control updates, illustrating the potential of the proposed framework for resilient networked cyber-physical systems.

From a methodological perspective, the polytopic framework offers a solid foundation for addressing future challenges in resilient cyber-physical systems. Promising research directions include distributed and multi-agent resilient control architectures, the integration of learning-based estimation techniques, secure event-triggered mechanisms, and their application to autonomous robotic and cyber-physical systems operating in increasingly dynamic and adversarial environments.

## Data Availability

No new data were created or analyzed in this study. Data sharing is not applicable to this article.
